# Comparison of machine learning clustering algorithms for detecting heterogeneity of treatment effect in acute respiratory distress syndrome: A secondary analysis of three randomised controlled trials

**DOI:** 10.1016/j.ebiom.2021.103697

**Published:** 2021-12-01

**Authors:** Pratik Sinha, Alexandra Spicer, Kevin L Delucchi, Daniel F McAuley, Carolyn S Calfee, Matthew M Churpek

**Affiliations:** a Division of Clinical and Translational Research, Division of Critical Care, Department of Anesthesia, Washington University School of Medicine, Saint Louis, MO; b Department of Medicine, University of Wisconsin- Madison, Madison, Wisconsin; c Department of Psychiatry and Behavioral Sciences; University of California, San Francisco; San Francisco, CA; d Wellcome-Wolfson Institute for Experimental Medicine, Queen's University Belfast; e Regional Intensive Care Unit, Royal Victoria Hospital, Belfast. Wellcome-Wolfson Institute for Experimental Medicine, Queen's University Belfast; f Department of Medicine, Division of Pulmonary, Critical Care, Allergy and Sleep Medicine; University of California, San Francisco; San Francisco, CA; g Department of Anesthesia; University of California, San Francisco; San Francisco, CA

**Keywords:** ARDS, RCTs, Clustering, machine learning, LCA, Heterogeneity of treatment effect

## Abstract

**Background:**

Heterogeneity in Acute Respiratory Distress Syndrome (ARDS), as a consequence of its non-specific definition, has led to a multitude of negative randomised controlled trials (RCTs). Investigators have sought to identify heterogeneity of treatment effect (HTE) in RCTs using clustering algorithms. We evaluated the proficiency of several commonly-used machine-learning algorithms to identify clusters where HTE may be detected.

**Methods:**

Five unsupervised: Latent class analysis (LCA), K-means, partition around medoids, hierarchical, and spectral clustering; and four supervised algorithms: model-based recursive partitioning, Causal Forest (CF), and X-learner with Random Forest (XL-RF) and Bayesian Additive Regression Trees were individually applied to three prior ARDS RCTs. Clinical data and research protein biomarkers were used as partitioning variables, with the latter excluded for secondary analyses. For a clustering schema, HTE was evaluated based on the interaction term of treatment group and cluster with day-90 mortality as the dependent variable.

**Findings:**

No single algorithm identified clusters with significant HTE in all three trials. LCA, XL-RF, and CF identified HTE most frequently (2/3 RCTs). Important partitioning variables in the unsupervised approaches were consistent across algorithms and RCTs. In supervised models, important partitioning variables varied between algorithms and across RCTs. In algorithms where clusters demonstrated HTE in the same trial, patients frequently interchanged clusters from treatment-benefit to treatment-harm clusters across algorithms. LCA aside, results from all other algorithms were subject to significant alteration in cluster composition and HTE with random seed change. Removing research biomarkers as partitioning variables greatly reduced the chances of detecting HTE across all algorithms.

**Interpretation:**

Machine-learning algorithms were inconsistent in their abilities to identify clusters with significant HTE. Protein biomarkers were essential in identifying clusters with HTE. Investigations using machine-learning approaches to identify clusters to seek HTE require cautious interpretation.

**Funding:**

NIGMS R35 GM142992 (PS), NHLBI R35 HL140026 (CSC); NIGMS R01 GM123193, Department of Defense W81XWH-21-1-0009, NIA R21 AG068720, NIDA R01 DA051464 (MMC)

**Research in context**

**Evidence before this study**

Heterogeneity subsumed within clinical critical care syndromes, such as acute respiratory distress syndrome (ARDS), has led to a plethora of “negative” clinical trials. To circumnavigate this heterogeneity, investigators are increasingly seeking heterogeneity of treatment effect (HTE) in novel phenotypes/subgroups, with several studies describing approaches to clustering using a multitude of clustering algorithms and data types. The best approach used for identifying clusters where HTE is detectable in secondary analysis of clinical trials, and their comparative benefits and limitations, is not known.

**Added value of this study**

In this study, we present secondary analyses of three randomised controlled trials (RCTs) of ARDS where we compare nine clustering algorithms (five unsupervised and four supervised) to seek HTE in the identified subgroups. We used a composite of clinical data and protein biomarkers as partitioning variables. Latent class analysis (LCA), causal forest and x-learner with random forest identified HTE in 2/3 of the trials. No single algorithm consistently identified clusters with HTE. LCA aside, most algorithms were highly susceptible to random seed changes leading to alteration of cluster composition and HTE detection. Protein biomarkers were essential partitioning variables for successful detection of HTE in the identified clusters.

**Implications of all available evidence**

Taken together with the wider literature, our findings reinforce the feasibility of detecting HTE in subgroups identified using machine learning algorithms. The inconsistencies observed with seed changes in some of the machine learning algorithms warrants cautious interpretation of such findings and mandates their prospective validation. It is highly probable that no single algorithm will work in all RCTs and future studies should focus on matching algorithms that are best suited to specific data structures and trial designs.

Alt-text: Unlabelled box

## 1 Introduction

Randomised controlled trials (RCTs) in the traditional paradigm of evidence-based medicine assume uniform treatment responses among all individuals. It is clear, however, that this assumption is invalid, and becomes more so with increasing heterogeneity in the study population[1,2]. In critical care, where therapies are frequently tested in non-specific clinical syndromes with broad diagnostic criteria, such as sepsis and acute respiratory distress syndrome (ARDS), numerous trials have failed to deliver successful therapies [3,4]. The central premise of precision or personalised medicine is to challenge this paradigm and focus on delivering the right therapies to the right patient by embracing the concept of heterogeneity of treatment effect (HTE). HTE is defined as a non-random, explainable variability in the direction and magnitude of treatment effect [5]. Conceptually, subgroup analyses in RCTs are the most common approach to evaluating HTE. However, performing multiple subgroup analyses in RCTs can result in false discoveries [6,7]. A more predictive, unbiased, and multivariable approach to HTE analyses has been proposed, as it avoids the use of single variables with arbitrary cut-offs to determine subgroups [8].

Increasingly, research groups are using machine learning (ML) approaches to identify subgroup within critical care syndromes with the hope of identifying HTE. For example, in ARDS, using latent class analysis (LCA), two subphenotypes of ARDS, with divergent biological features, clinical characteristics and outcomes, have been consistently described across five RCTs [9,12]. In three of these RCTs, the phenotypes showed differential responses to randomised interventions. In sepsis, using k-means clustering, investigators have identified subgroups that showed HTE [13]. Others have proposed using supervised ML algorithms to determine characteristics associated with HTE [14,15]. The optimal approach to finding clusters where HTE is observable, however, remains unknown.

The main objective of this exploratory study was to ascertain the optimal ML algorithm to consistently identify clusters with HTE. Frequently, approaches that combine protein biomarker data with clinical data are used as partitioning variables for subgroup discovery [8]. The importance of protein biomarkers in identifying clusters where HTE is observed remains uncertain. A secondary objective, therefore, was to test whether the detection of HTE was predicated on the inclusion of protein biomarkers as partitioning-variables.

## 2 Methods

### 2.1 Overview

**Fig. 1:**
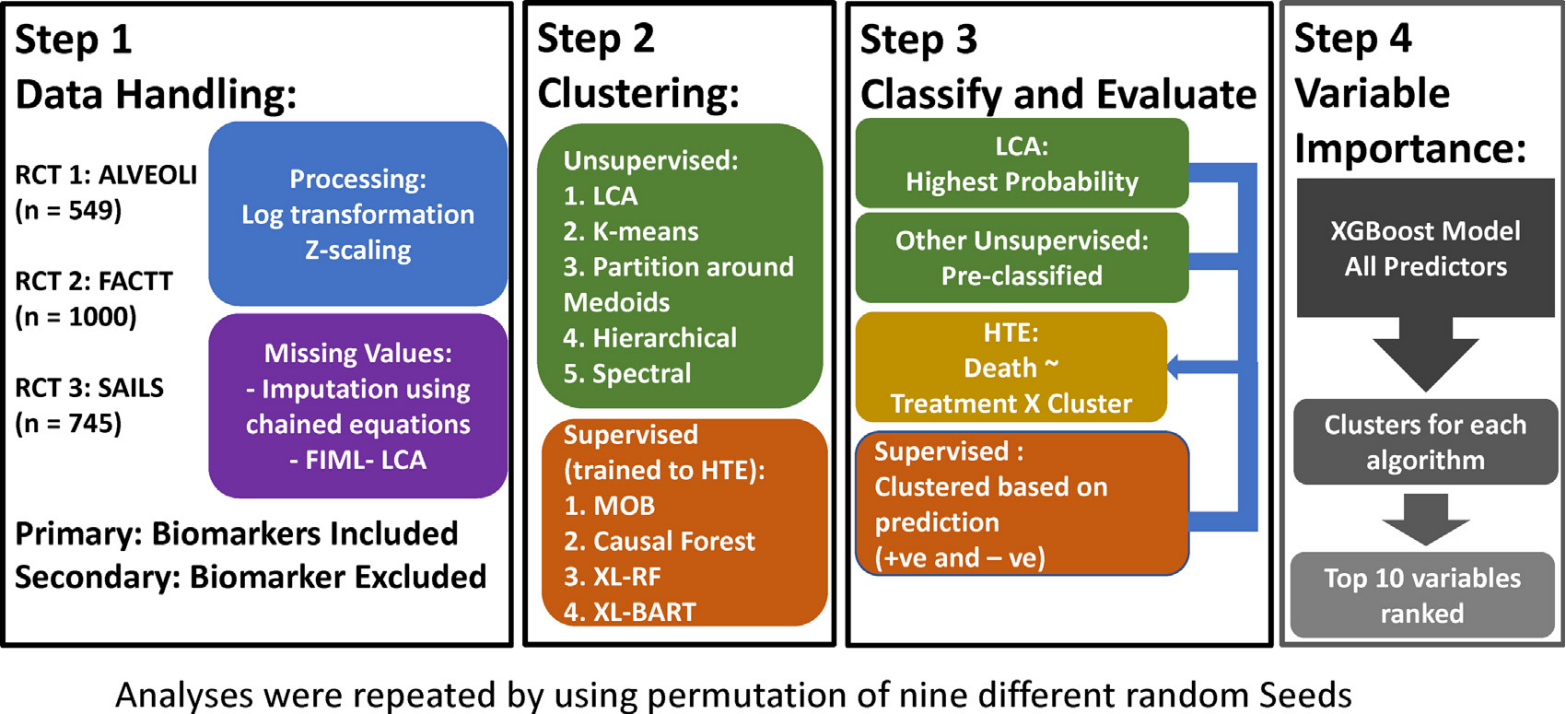
**Overview of the analysis plan and study design**. ALVEOLI = Assessment of Low Tidal Volume and Elevated End-Expiratory Pressure to Obviate Lung Injury, FACTT = Fluids and Catheters Treatment Trial, SAILS = Statins for Acutely Injured Lungs from Sepsis, FIML = Full information maximum likelihood. LCA = Latent class analysis, MOB = model based recursive partitioning, XL-RF = X-learner with Random Forest (RF); XL-BART = Bayesian Additive Regression Trees (BART). HTE = Heterogeneity of treatment effect.

The overview of the analysis plan is summarised in Fig. 1. Briefly, the performance of each of the nine clustering algorithm was evaluated independently in secondary analyses of three prior ARDS RCTs. Baseline variables pertaining to demographics, vital signs, ventilatory metrics, clinical laboratory measurements, and research protein biomarkers served as predictors for each algorithm (**Table S1**). For each algorithm, once patients were classified into clusters, HTE for 90-day mortality was sought, and variable importance for cluster classification was estimated. For secondary analyses, we repeated the above analyses by 1) generating permutations of random seed initiation for relevant algorithms to assess their stability; 2) excluding the protein biomarkers as predictor variables.

### 2.2 Study Population

Data from three ARDS RCTs conducted by the National Heart, Lung and Blood Institute's ARDS-Network were used. Permission to conduct these analyses was provided by BioLINCC. ALVEOLI (n=549) tested the efficacy of high positive end-expiratory pressure (PEEP) compared to usual care/lower PEEP [16]. FACTT (n=1000) tested the efficacy of conservative versus liberal fluid management strategies [17]. SAILS (n=745) tested the efficacy of rosuvastatin versus placebo [18]. Specifically, these three trials were selected because the clinical data and biomarker availability, timing of recruitment, and inclusion criteria were relatively uniform across all three RCTs (**Table S2**). The use of these three RCTs would enable algorithmic performance evaluation in the same clinical syndrome and independent of variances in partitioning variables. In SAILS, in addition to ARDS diagnosis, the inclusion criteria mandated that subjects had a known/suspected infection and met systemic inflammatory response syndrome criteria. Partitioning variables in the clustering algorithms were collected prior to randomisation. Biospecimens for protein biomarkers were collected at enrolment. Assay procedures are described in prior publications [9,11,12].

### 2.3 Clustering algorithms

The clustering algorithms selected for evaluation were either those commonly described in the medical literature or prominent in the data science literature [19]. The algorithms used for unsupervised clustering were categorised as 1) distance-based: K-means, partitioning around medoids (PAM), hierarchical clustering (HC), and spectral clustering (SC); 2) probability-based: latent class analysis (LCA). These algorithms were implemented agnostic to outcome and treatment group allocation.

All non-normally distributed continuous predictor variables were log-transformed. For algorithms that required complete data, missing values were replaced by single imputation with chained equations (*MICE* package in R) and the same imputed data was used in all algorithms. A summary of the missing values can be found in **Table S3**. For unsupervised methods, all continuous variables were z-scaled prior to being used in the algorithms (mean = 0, standard deviation = +/-1). For distance-based clustering, Euclidean distance was used to determine clusters.

Two to five clusters were compared in the unsupervised approaches. Optimal number of clusters was determined using an unbiased and automated approach for the distance-based algorithms called consensus clustering. In consensus clustering, each algorithm was run 1000-times with various combinations of variables, observations, and multiple random starting locations for each run. The cumulative results for each clustering solution are captured in a consensus matrix. The distribution of the proportion of ambiguous clustering (PAC) on the cumulative distribution function plot was used to identify the most stable clustering solution using the *ConsensusClusterPlus* package in R. For LCA, the model comprising the optimal number of classes was selected using the Vuong-Lo-Mendel-Rubin test (primary selection criteria), Bayesian information criteria (BIC), entropy, and the size of the smallest class [20]. Each model was run with up to 1000 random starts, and models were only considered for evaluation if the maximum likelihood ratio replicated in at least 20 starts.

The supervised clustering algorithms were trained directly to predict differential treatment responses using outcome and treatment group allocation as the dependent variables. The four algorithms used were: model based recursive partitioning (MOB), Causal Forest (CF), and X-learner with Random Forest (XL-RF) and Bayesian Additive Regression Trees (XL-BART). For all algorithms, out-of-sample prediction for individual treatment effect (ITE) was generated per observation. Patients were classified into one of two clusters, based on the whether the ITE coefficient was positive or negative. Details of all algorithms and procedures used are summarised in the supplement.

### 2.4 Statistical analysis

For both the supervised and unsupervised approaches, once the optimal number of clusters were identified and observations classified into clusters, HTE was tested using logistic regression models, where 90-day mortality served as the dependent variable and the interaction term of clusters and treatment group was the independent variables. P-values for the interaction term were generated using the ANOVA likelihood ratio test and a significant interaction term (p<0.05) was defined as success for HTE. Odds ratio for heterogeneity of treatment effect in the clusters was generated for each algorithm (odds ratio > 1 was associated with harm and < 1 with benefit). Chi-squared test was used to test differences in outcome between the identified clusters.

### 2.5 Variable importance

To determine the most contributory partitioning variables for cluster identification, for each algorithm, gradient boosted machine models (XGBoost) were trained to classify the identified clusters using the same partitioning-variables as predictors in the model (**Table S1**). The gain in accuracy in cluster classification with the addition of a variable was computed and cumulatively tallied for each XGBoost model to obtain the relative importance of each variables per algorithm.

### 2.6 Sensitivity analyses

ML algorithms are subject to instability with perturbations in the random initialisation seed (the starting point from which algorithms are generated). To test the reproducibility of the algorithms, we repeated the above analyses with random seed iterations for all approaches (except LCA, where models were only selected if the maximum likelihood was reproduced in multiple initiation seeds). Nine further runs were performed, each with a new random seed for single imputation and the clustering algorithms. Inter-run similarities in clusters identified across the 10 runs were evaluated using the Adjusted Rand Index (ARI).

Finally, to test the importance of biomarkers for identifying clusters where HTE was observed, we repeated the above analyses by excluding protein biomarkers as predictors.

All clustering approaches, except LCA, were performed on RStudio version 1.1.453 using R version 4.0.1. LCA was performed using MPlus version 8.5.

### 2.7 Ethics statement

Local institutional review board granted a consent-waiver for the use of de-identified trial data for research purposes.

### 2.8 Role of funders

Funders of the study had no role in study design, data collection, data analysis, data interpretation, or writing of the report.

## 3 Results

**Fig. 2:**
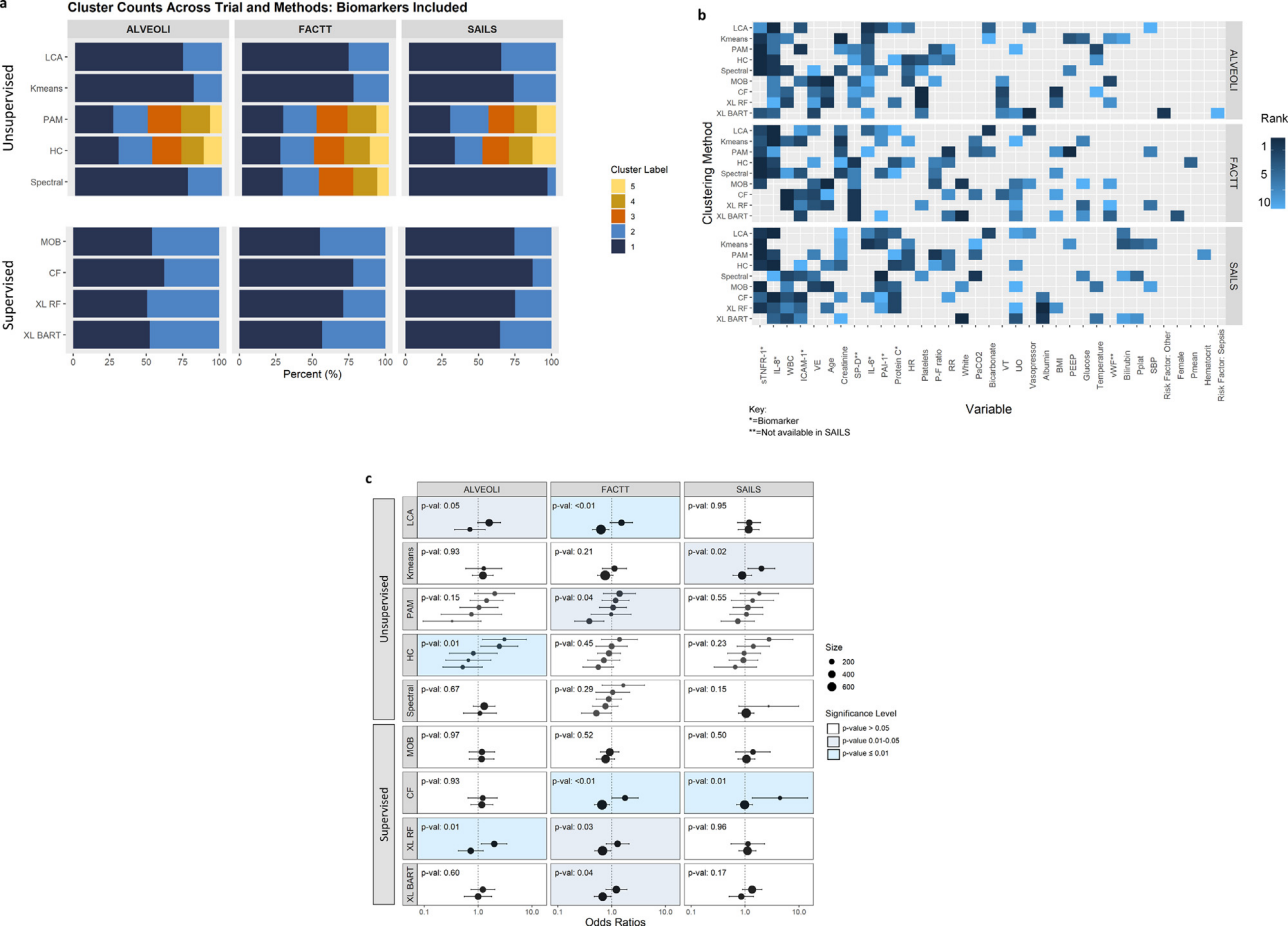
**Summary of the primary clustering analyses in the three trials**. ALVEOLI = Assessment of Low Tidal Volume and Elevated End-Expiratory Pressure to Obviate Lung Injury (N = 549), FACTT = Fluids and Catheters Treatment Trial (N = 1000), SAILS = Statins for Acutely Injured Lungs from Sepsis (N = 745). LCA = Latent class analysis, PAM = partitioning around medoids, HC = Hierarchical clustering, MOB = model based recursive partitioning, CF = Causal forest, XL-RF = X-learner with Random Forest (RF); XL-BART = Bayesian Additive Regression Trees (BART). **Panel 2a: Optimal number of clusters and proportions of patients in each clusters per algorithm** (The colours are representative of the size of the clusters)**. Panel 2b: Top 10 variable importance for each clustering algorithm.** sTNFR-1 = Soluble tumour-necrosis factor receptor-1, IL-8 = Interleukin-8, WBC = White blood cell count, ICAM-1 = Intercellular adhesion molecule-1, VE = Minute ventilation, SP-D = Surfactant protein-D, IL-6 = Interleukin-6, PAI-1 = Plasminogen activator inhibitor-1, HR = Heart rate, P:F ratio = PaO_2_/FiO_2_, White (race), VT = Tidal volume, UO = Urine output, BMI = Body mass index, PEEP = = Positive end-expiratory pressure, vWF = Von Willebrand Factor, Pplat = Plateau pressure, SBP = Systolic blood pressure, Pmean = Mean airway pressure. **Panel 2c: Odds ratio for heterogeneity of treatment effect in clusters for each algorithm** (odds ratio > 1 was associated with harm, p-value represents the significance of the coefficient of the interaction term of randomised intervention and clusters in a logistic regression model with mortality at day 90 as the dependent variable; P-values were generated for the interaction term using the ANOVA likelihood ratio test)**.**

The baseline characteristics of the three RCTs and the predictor variables are summarised in **Table S1**. Among the unsupervised clustering approaches, the number of clusters identified across the cohorts were largely consistent across the three trials. LCA and k-means consistently identified two clusters, whereas PAM and HC identified five clusters (Fig. 2**a**). Spectral identified two classes in ALVEOLI and SAILS, and five classes in FACTT. The differences in mortality at day-90 between the identified clusters for each algorithm in each trial is summarised in Table 1.

**Table 1:** Mortality at day 90 in the three trials stratified by the clusters identified by each algorithm. P-values represent chi-squared tests between cluster category and death.

% Died in each Cluster								
Trial	Method		Cluster 1	Cluster 2	Cluster 3	Cluster 4	Cluster 5	p-value
**ALVEOLI**	Unsupervised	LCA	20.0	46.2	–	–	–	< 0.01
		K-means	23.0	43.8	–	–	–	< 0.01
		PAM	20.3	24.0	46.4	10.2	43.2	< 0.01
		HC	21.5	13.5	30.3	34.5	50.7	< 0.01
		Spectral	22.0	43.3	–	–	–	< 0.01
	Supervised	MOB	25.0	25.0	–	–	–	0.31
		CF	27.8	29.2	–	–	–	0.65
		XL RF	27.0	27.0	–	–	–	0.99
		XL BART	19.4	35.2	–	–	–	< 0.01
**FACTT**	Unsupervised	LCA	22.1	45.1	–	–	–	< 0.01
		K-means	24.4	41.1	–	–	–	< 0.01
		PAM	14.2	25.1	31.8	38.3	52.9	< 0.01
		HC	42.5	18.3	20.3	29.9	28.5	< 0.01
		Spectral	18.1	30.4	36.1	23.0	47.4	< 0.01
	Supervised	MOB	25.0	32.6	–	–	–	0.01
		CF	26.9	33.6	–	–	–	0.06
		XL RF	26.1	33.9	–	–	–	0.02
		XL BART	32.4	23.1	–	–	–	< 0.01
**SAILS**	Unsupervised	LCA	21.4	37.5	–	–	–	< 0.01
		K-means	24.4	34.7	–	–	–	0.01
		PAM	23.9	19.6	38.2	20.9	43.8	< 0.01
		HC	22.4	33.3	44.0	20.0	18.6	< 0.01
		Spectral	26.6	40.5	–	–	–	0.07
	Supervised	MOB	30.4	18.5	–	–	–	< 0.01
		CF	28.3	20.8	–	–	–	0.16
		XL RF	29.9	19.9	–	–	–	0.01
		XL BART	25.1	31.7	–	–	–	0.06

ALVEOLI = Assessment of Low Tidal Volume and Elevated End-Expiratory Pressure to Obviate Lung Injury (N = 549), FACTT = Fluids and Catheters Treatment Trial (N = 1000), SAILS = Statins for Acutely Injured Lungs from Sepsis (N = 745). LCA = Latent class analysis, PAM = partitioning around medoids, HC = Hierarchical clustering, MOB = model based recursive partitioning, CF = Causal Forest, XL-RF = X-learner with Random Forest (RF); XL-BART = Bayesian Additive Regression Trees (BART).

Among the unsupervised methods, pro-inflammatory biomarkers, such as interleukin (IL)-8, IL-6 and soluble tumour-necrosis factor receptor (sTNFR)-1, were prominently and consistently featured as important variables (Fig. 2**b**). In the supervised approaches, however, no discernible patterns were observable across the three trials. Even within a trial, the pattern of variable importance differed between the supervised algorithms.

Significant HTE interaction with clusters was observed using LCA and X-learner RF clusters in ALVEOLI and FACTT (Fig. 2**c**). Using CF, significant HTE were observed in FACTT and SAILS. Using K-means, PAM, HC and XL-BART, significant HTE interactions were only observed in one out of the three trials. No significant HTE interactions were observed in clusters identified by either spectral clustering or MOB. In clusters where significant HTE interactions were observed in the same trial, there were substantial differences in patient composition of clusters across the algorithms (**Fig. S1**). For example, in FACTT, where LCA and XL-BART both identified two clusters with significant HTE, more than half the patients from the increased-mortality cluster in XL-BART crossed over to the lower mortality cluster in LCA and vice versa.

**Fig. 3:**
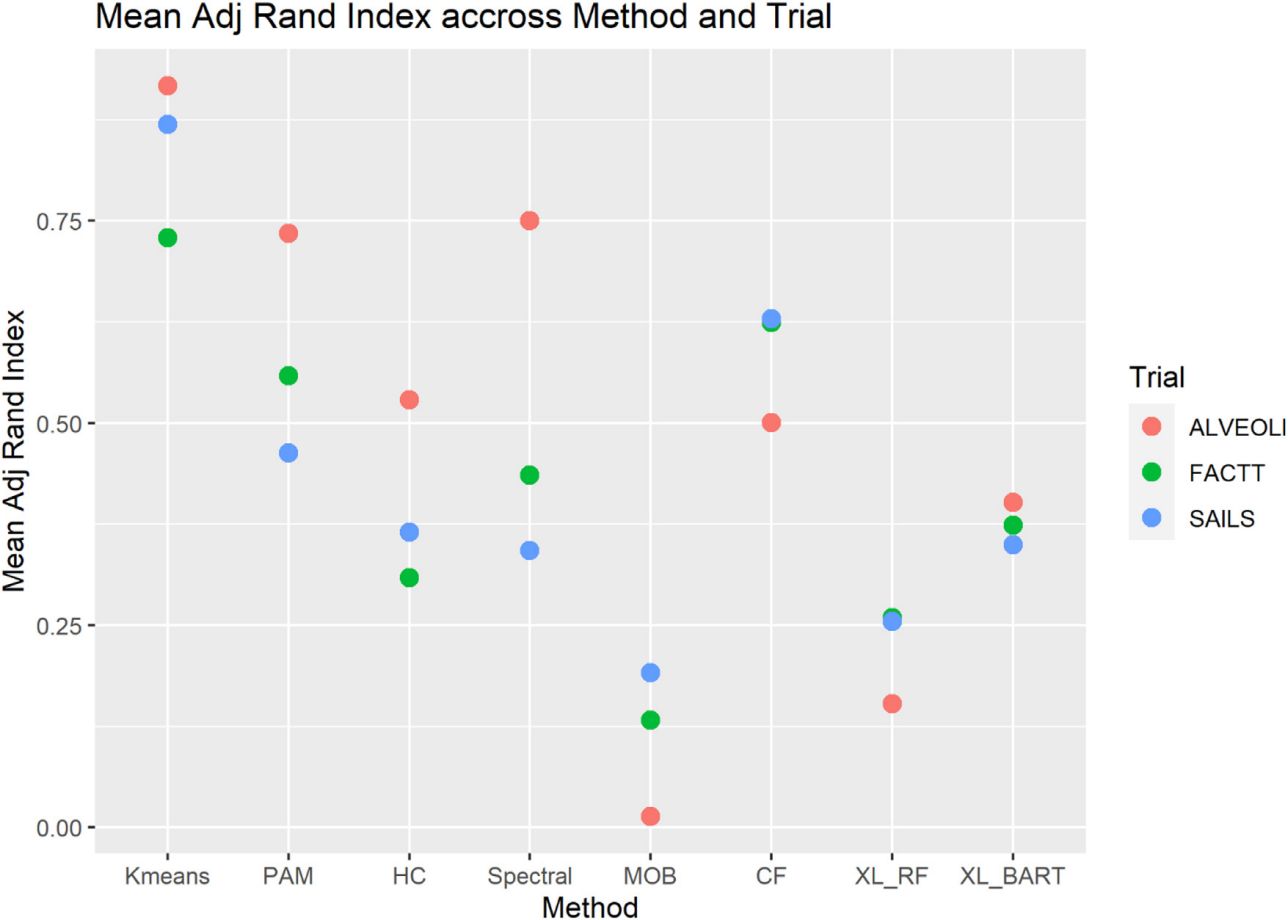
**Mean adjusted rand index score across the random seed runs for each algorithm in each trial** (an adjusted rand index score approaching 1 would represent almost complete agreement between cluster composition in the 10 random seed changes runs, whereas, a score of 0 represents almost no agreement). PAM = partitioning around medoids, HC = Hierarchical clustering, MOB = model based recursive partitioning, CF = Causal forest, XL-RF = X-learner with Random Forest (RF); XL-BART = Bayesian Additive Regression Trees (BART).

In all trials, with both the supervised and unsupervised algorithms, permutations in random seed initialisation in sequential runs led to several HTE interaction terms no longer being significant despite the same number of clusters being identified. In ALVEOLI, in 10 consecutive runs, significant HTE interactions were observed only in 2 of 10 runs in HC, 3 of 10 runs in XL-RF and 1 of 10 runs in MOB (**Fig. S2a)**. In FACTT, significant HTE was more consistently observed in the supervised approaches (CF and XL-BART; 6 and 4 out of 10 runs respectively) but were inconsistent in the unsupervised approaches (**Figure S2b**). In SAILS, the findings of HTE were consistent in k-means and XL-BART, however, in CF and XL-RF HTE was observed in only 1 out of 10 runs (**Fig. S2c**). The mean Adjusted Rand index, a measure of pairwise similarity, for each trial varied widely in each algorithm, and its value was generally low throughout, indicating poor overlap between clusters identified using the various random seed runs for the same algorithm in the same trial cohort (Fig. 3).

**Fig. 4:**
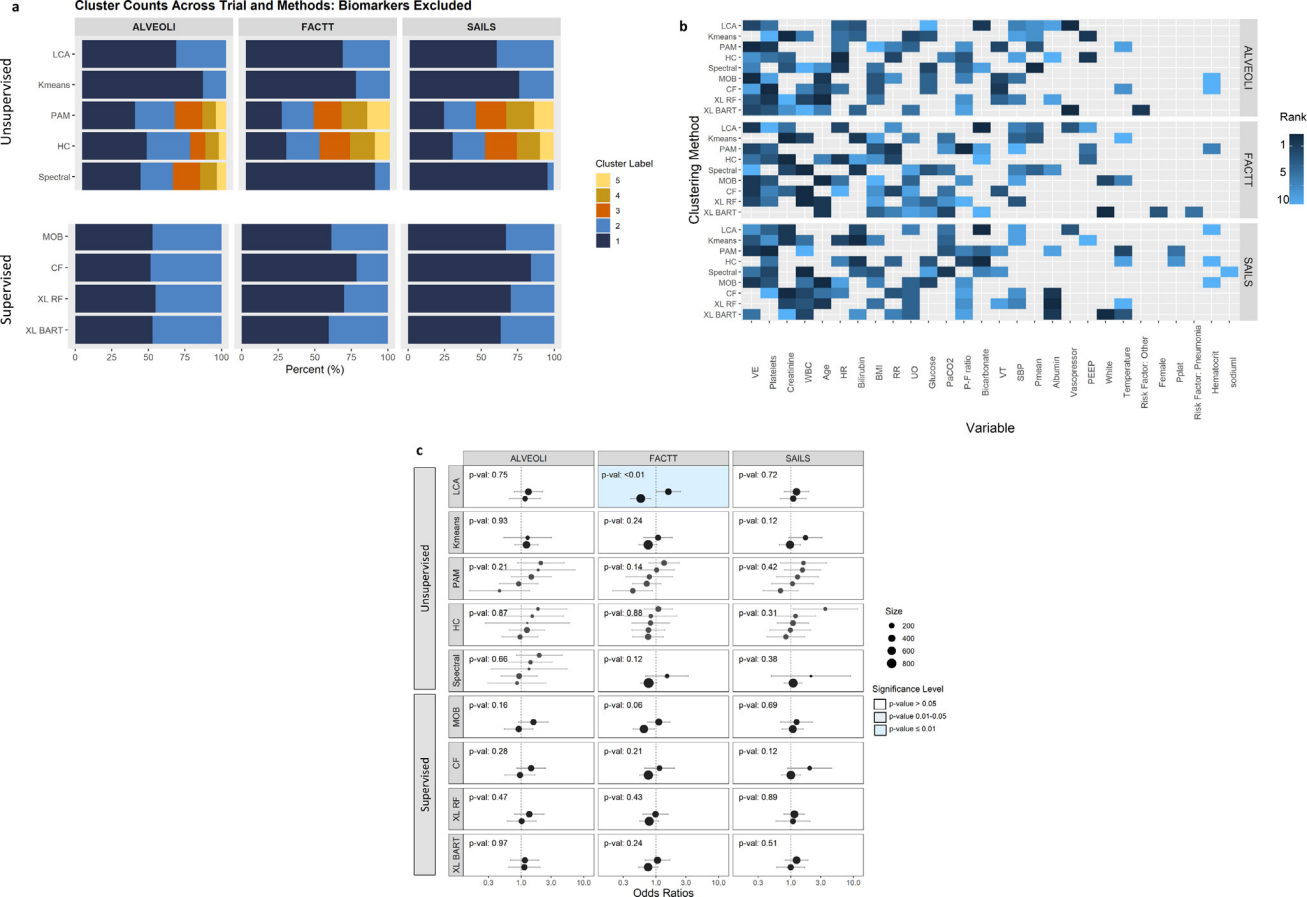
**Summary of the secondary clustering analyses in the three trials where protein biomarker data were excluded as partitioning variables.** ALVEOLI = Assessment of Low Tidal Volume and Elevated End-Expiratory Pressure to Obviate Lung Injury (N = 549), FACTT = Fluids and Catheters Treatment Trial (N = 1000), SAILS = Statins for Acutely Injured Lungs from Sepsis (N = 745). LCA = Latent class analysis, PAM = partitioning around medoids, HC = Hierarchical clustering, MOB = model based recursive partitioning, CF = Causal forest, XL-RF = X-learner with Random Forest (RF); XL-BART = Bayesian Additive Regression Trees (BART). **Panel 4a: Optimal number of clusters and proportions of patients in each cluster per algorithm** (The colours are representative of the size of the clusters)**. Panel 4b: Top 10 variable importance for each clustering algorithm.** WBC = White blood cell count, VE = Minute ventilation, HR = Heart rate, P:F ratio = PaO_2_/FiO_2_, White (race), VT = Tidal volume, UO = Urine output, BMI = Body mass index, PEEP = = Positive end-expiratory pressure, Pplat = Plateau pressure, SBP = Systolic blood pressure, Pmean = Mean airway pressure. **Panel 4c: Odds ratio for heterogeneity of treatment effect in clusters for each algorithm** (odds ratio > 1 was associated with harm, p-value represents the significance of the coefficient of the interaction term of randomised intervention and clusters in a logistic regression model with mortality at day 90 as the dependent variable; P-values were generated for the interaction term using the ANOVA likelihood ratio test).

When the above analyses were repeated with protein biomarkers excluded, as partitioning variables the optimal number of clusters across each algorithm mostly remained the same, albeit, the proportion of observations in each cluster were markedly different (Fig. 4**a**). Important predictor variables across supervised and unsupervised approaches were more similar, however, there remained notable heterogeneity across the various algorithms (Fig. 4**b**). Among all the clustering algorithms, without protein biomarker variables, significant HTE interaction was only observed in clusters derived using LCA in the FACTT trial (Fig. 4**c)**.

## 4 Discussion

In this study, we found wide variations between ML algorithms in their abilities to identify clusters in which significant HTE was observed. Even among algorithms where significant HTE was observed, changing the initiation seed frequently led to the interaction terms no longer being significant. Further, in most clustering algorithms, seed changes also led to heterogeneity in the cluster composition across the different runs. Additionally, as evidenced by the near ubiquitous lack of significant HTE in their absence, research protein biomarkers were critical to identifying clusters with HTE. Taken together, our findings suggest the ability to identify clusters with HTE in RCTs is dependent on the clustering algorithm and partitioning variables used, and no single approach consistently identified clusters with HTE. Further, the inherent algorithmic instability of several approaches mandates caution when interpreting research based on these algorithms.

The relative parity in success of unsupervised and supervised approaches in detecting HTE is unexpected. It is worth reiterating that for unsupervised approaches, outcome data and treatment allocation were excluded from the algorithms. In contrast, supervised approaches were trained directly to detect HTE, and therefore, we anticipated these approaches to out-perform unsupervised ones. Traditionally, unsupervised learning approaches are used for discovering underlying structure in unlabelled data [21,22]. Predominantly, such approaches have been used for biomarker discovery, however, several recent studies have shown that in secondary analyses of RCTs, clusters identified using unsupervised methods can lead to discoveries of differential treatment responses [9,11]. Our findings suggest that there may be validity in using unsupervised approaches to identify clusters with HTE, although some algorithms (e.g., LCA) performed better than others.

Another potential reason for the relative underperformance of the supervised methods in our study was that we coerced coefficients of individual treatment effect (ITE) into dichotomous subgroups to make them comparable to the unsupervised clustering algorithms, a process that is known to cause information loss [23]. Future investigations may need to focus more on maximising ITE, rather than using more traditional approaches of subgroup / cluster HTE analyses, specifically when comparing supervised methods to one another.

Two potential mechanism that can lead to the discovery of HTE have been described, 1) prognostic enrichment: whereby the outcome of interest differs between the identified clusters; 2) predictive enrichment: which seeks to identify patients that are most likely to benefit from a therapy. By definition, given that the models are trained to seek treatment responsive groups, supervised approaches are focused towards predictive enrichment. The similarity in outcomes between clusters, and differences in the most important variables, for the supervised algorithms between RCTs observed in our study this. In contrast, the most important variables in the unsupervised approaches that identified clusters with HTE were largely overlapping across RCTs. Which, coupled with the large differences in mortality, is highly suggestive of prognostic enrichment. Prognostic enrichment approaches, while more generalisable across trials, may be non-specific to the intervention.

Our findings were also notable for disparities in the variable importance and cluster composition between algorithms in the same trial. This finding was even notable among clusters where HTE was identifiable in the same trial, suggesting that within populations there may be several different subgroups of patients where HTE is identifiable or that there are a number of patients where their treatment effects are uncertain. In FACTT, among the unsupervised approaches, HTE was detectable using LCA and PAM, and clusters were stratified using IL-8, IL-6, sTNFR-1, and creatinine. Among the supervised approaches, HTE was detectable in CF, X-learner RF and BART, and patient clusters were stratified predominantly using surfactant protein-D, white cell count, intercellular adhesion molecule-1 and minute ventilation. Coupled with the high crossover of patients from the treatment-benefit to treatment-harm cluster and vice versa, our findings suggest that there may be numerous sub-populations where HTE may be detectable within the same trial population. Even when the same computational framework, such as X-learner, was used, the algorithm used to cluster (RF or BART) had a profound impact on the composition of the clusters as evidenced by crossover of patients between clusters identified using the two approaches. These findings further reinforce the necessity to prospectively and externally validate HTE that has been discovered in secondary analyses of RCTs and, as such, this has also been emphasised in a recent multidisciplinary expert panel statement for studies using predictive approaches to seek HTE [24].

The inherent instability of several of the algorithms to random seed makes interpretation of such analyses challenging. Random seed perturbations leading to algorithmic instability have been described in other machine-learning approaches [25,26]. ^[pre-print]^ However, this phenomenon remains underappreciated in the medical literature. Inadvertently, investigators may misinterpret local maxima as the global maxima (i.e., erroneously accept a suboptimal solution). In algorithms where seed instability is known to be a factor, investigators should demonstrate the robustness of their findings to seed iterations. To that end, it is noteworthy that in LCA, a probabilistic algorithm for determining classes, where the model parameters are subject to statistical assumptions and hypothesis testing is feasible [20], cluster instability to seed perturbations was not a factor. This consistency of subgroup identification may partly explain why LCA was one of the most successful methods at identifying clusters with HTE.

Alongside seed instability, the algorithmic procedures being concealed in a black box, are some of the greatest barriers to real-life clinical implementation of many machine learning algorithms. Black box algorithms generate predictions absent of contemporaneous justification. From the standpoint of traditional medical practices, a lack of understanding of the “how” of the algorithm can be counter-intuitive and a barrier to adopting machine-learning clinical decision-making [27]. ^[pre-print]^ The first steps towards implementation of black-box algorithms is to establish its superiority to simpler and / or more intuitive models such as logistic regression or LCA. Next, as with any other modelling, the consistency and robustness of its prediction must be demonstrated in a prospective setting. Once the utility and validity of such black-box models has been established, efforts need to made towards creating models that accompany concise and clear explanation for the prediction at an individual patient level. The burgeoning field of explainable machine learning endeavours to create such models by either presenting the most important variables driving the prediction or an interpretable translation of the model design or both [28]. ^[pre-print],^ [29] Based on these factors, it is incumbent for investigators to determine the plausibility of the explanation for the generated prediction and to establish validity from a scientific / biological standpoint.

Inclusion of research protein biomarkers, such as IL-6, IL-8, sTNFR-1 and surfactant protein-D, were crucial in identifying clusters with HTE. These variables consistently featured among the most important variables and computationally explains why their absence led to treatment interactions no longer being observed. It may be that the biological characteristics in clusters derived using these variables cannot be captured simply using routinely gathered clinical data or by the clinical data collected at baseline in the included trials. Therefore, when biomarkers are used for partitioning populations, we hypothesise that the resultant clusters are more likely to be pathophysiologically divergent, increasing the possibilities of divergent treatment responses [10]. Our study's findings strongly suggest that inclusion of novel biological data can improve the chances of discovering clusters with divergent outcomes and treatment responses in ARDS; how generalisable this finding is to other heterogenous syndromes remains to be tested.

This study has several strengths. Most notably, we used several machine learning approaches including those that are most widely used and many that are cutting edge. Throughout, we have used objective and automated approaches to selecting the optimal number of clusters. We also evaluated the models in three independent RCTs, adding validity to the findings.

This study also has limitations. Subsumed within our primary hypothesis was the assumption that HTE is present in all three RCTs. However, it is probable this may not be the case. For example, in SAILS, given how infrequently HTE was observed, and that observed HTE was in the context of largely imbalanced cluster sizes, it is conceivable that “true” HTE is not present in this population, however, such a discovery was attributed as unsuccessful in our study. To that end, when translating the findings of HTE analyses in RCTs, the ground truth will seldom, if ever, be known. Therefore, caution must be exercised when designing such studies in order to mitigate bias associated with overfitted models that are specific to the training dataset. When reporting findings of such analyses, measure of robustness should be incorporated, and agreement between multiple orthogonal approaches should be considered to verify the findings in instances where there is uncertainty on the validity of the observed HTE. It is also worth reiterating that, until tested prospectively, the validity of the observed HTE in secondary analyses, such those presented in this study, remains unproven. Additionally, these results are secondary analyses of RCTs of a single clinical syndrome. The performance of these algorithms prospectively and beyond ARDS is unknown. Further, these trials can all be considered as “small” data. It is unclear whether in analyses of larger RCTs, the observed seed instability of the algorithms and critical importance of protein biomarkers remain valid. Based on the available data and the presented analyses, we have not been able to determine which of these algorithms performed best at detecting HTE. It may be that the best algorithm to use may depend on the data structure and size and the quality of the available predictor variables for use in the models. In future studies, we hope to develop an investigative pipeline that may help researchers match their trial data characteristics to the most appropriate algorithm for detecting HTE.

In conclusion, machine learning algorithms were inconsistent in their abilities to identify clusters with significant HTE in secondary analyses of RCTs. Several of the clustering algorithms were susceptible to significant instability with random seed initiations. This stability of algorithms to seed changes should be factored into future evaluation of such studies. In these populations, inclusion of research protein biomarkers as partitioning variables greatly enhanced the ability of the algorithms to identify clusters with HTE. Further studies are needed to establish machine learning pipelines that can robustly and consistently identify either at a subgroup or individual level those that will benefit from therapies in RCTs.

## 5 Contributors

CSC, MMC, PS and AS contributed to the initial study design. MMC, PS and AS were involved in planning, conduct and reporting of the work described in the study. KLD contributed with study design and analysis of the study. CSC, DFMA and PS contributed to the development of the datasets used for preparation and execution of the studies presented in the manuscript. PS developed the first draft. All authors contributed to the final preparation of the manuscript. PS and MMC accept full responsibility for the work and had access to the data and controlled the decision to publish.

## 6 Data Sharing

The non-biomarker data were sourced from publicly-available repositories. Requests for sharing of protein biomarker data will be evaluated on an individual basis.

## Declaration of Competing Interest

Dr Sinha received institutional grants from the National Institute of Health (NIH), Ms Spicer received institutional grant funding from the NIH and Department of Defense. Prof Delucchi declares no relevant conflicts of interest. Prof McAuley received institutional grants from the NIHR, Innovate UK, MRC, Novavax, Northern Ireland HSC R&D Division, Wellcome Trust; royalties/licenses from Queen's University Belfast for a patent of novel treatment for inflammatory diseases (USB962032); consulting fees from Bayer, GlaxoSmithKline, Boehringer Ingelhelm, Novartis, Eli Lilly, SOBI; payment from GlaxoSmithKline as an educational seminary speaker; travel support from Vir Biotechnology Inc and Faron Pharmaceuticals as a member of Data Safety Monitoring Board. His spouse received consultancy fees from Insmed and payments for participation in grant review panel from California Institute for Regenerative Medicine. Prof Calfee received institutional grants from the NIH, Bayer, Roche-Genentech, Quantum Leap Healthcare Collaborative; and personal fees from Quark, Vasomune, and Gen1e Life Science. Dr Churpek received institutional grants from the NIH, Department of Defense, and EarlySense.

## Appendix Supplementary materials

Image, application 1

Image, application 2

Image, application 3
