# Supplementary material for: Comparison of machine learning clustering algorithms for detecting heterogeneity of treatment effect in acute respiratory distress syndrome: A secondary analysis of three randomised controlled trials
Source: eBioMedicine. 2021 Dec 1;74:103697. doi: 10.1016/j.ebiom.2021.103697 (PMC8645454; doi:10.1016/j.ebiom.2021.103697)
Supplement: Supplementary file 1 [file mmc1.docx]

**Comparison of Machine Learning Clustering Algorithms for Detecting Heterogeneity of Treatment Effect in Acute Respiratory Distress Syndrome: A Secondary Analyses of Three Randomised Controlled Trials**

Pratik Sinha MB ChB PhD^1^, Alexandra Spicer BS^2^, Kevin L Delucchi PhD^3^, Daniel F McAuley MD MBBS^4,5^, Carolyn S Calfee MD MAS^6,7^, and Matthew M Churpek MD MPH PhD^2^

**Affiliations:**

1 Division of Clinical and Translational Research, Division of Critical Care, Department of Anesthesia, Washington University School of Medicine, Saint Louis, MO Pratik Sinha Assistant Professor.

2 Department of Medicine, University of Wisconsin- Madison, Madison, Wisconsin

3 Department of Psychiatry and Behavioral Sciences; University of California, San Francisco; San Francisco, CA

4 Wellcome-Wolfson Institute for Experimental Medicine, Queen’s University Belfast

5 Regional Intensive Care Unit, Royal Victoria Hospital, Belfast. Wellcome-Wolfson Institute for Experimental Medicine, Queen’s University Belfast

6 Department of Medicine, Division of Pulmonary, Critical Care, Allergy and Sleep Medicine; University of California, San Francisco; San Francisco, CA

7 Department of Anesthesia; University of California, San Francisco; San Francisco, CA

**Corresponding Author:** Dr. Pratik Sinha

660 S. Euclid Ave, Campus Box 8054

St. Louis, MO 63110

Ph: 314-273-3461; email: [p.sinha@wustl.edu](mailto:p.sinha@wustl.edu)

**Supplementary Material**

**Methods**

**Study Population**

All three randomised control trials (RCTs) used in these analyses were conducted by National heart, lung and blood institute’s acute respiratory distress syndrome (ARDS) Network. The RCTs tested interventions in patients with ARDS that were intubated and receiving mechanical ventilation at the time of intervention. The Assessment of Low Tidal Volume and Elevated End-Expiratory Pressure to Obviate Lung Injury (ALVEOLI) trial, was a multicentre trial that tested the efficacy of higher positive end-expiratory pressure (PEEP) versus usual care/lower PEEP.^1^ The study recruited patients between October 1999 and April 2002. In total 549 patients were recruited, with 273 patients in the control arm and 276 in the treatment arm. The Fluids and Catheters Treatment Trial (FACTT) was a multicentre RCT that tested the efficacy of a conservative fluid management strategy versus liberal fluid management (or usual care).^2^ The study enrolled patients between June 2000 and October 2004, and in total, 1000 patients were enrolled with 503 in the treatment arm and 497 in the liberal fluid arm. Finally, in the Statins for Acutely Injured Lungs from Sepsis (SAILS) trial, patients were randomised to either receive Rosuvastatin or placebo. The study enrolled patients between March 2010 and September 2013 and recruited 745 patients with 379 assigned to the statin arm and 366 in the placebo arm.^3^ In all three trials, no differences were observed in the treatment arm compared to the control arm in the primary outcome (mortality at day 60). The major inclusion and exclusion criteria for the studies are summarised in **Table S1** and further details can be found in the original studies.

**Data Processing**

Data from either the time of enrolment or 24-hours pre-enrolment were used for the clustering analysis. Variables were selected as predictors based on their association with ARDS pathogenesis, severity or severity of critical illness. The protein biomarkers were, similarly, selected as predictors due to their association with severity of lung injury. The predictor variables used in the modelling are summarised in **Table S2**.

For the algorithms that necessitated complete data, single imputation using chained equations was performed using the *R package “MICE.”* This algorithm fills in missing values by first replacing missing values with the mean, iteratively restoring missingness in one variable at a time and filling in these missing values with predictions generated by a linear or logistic regression model using all other variables as the independent predictors. Details of the missing predictor variables in each trial cohort can be found in **Table S3**.

**Clustering Algorithms**

**Unsupervised Approaches**

Unsupervised clustering methods group observations based on similarities detected within the distribution of the predictor variables. For all clustering algorithms, 2-5 cluster solutions were evaluated and information pertaining to outcomes and treatment were excluded. Non-normally distributed data were log-transformed. Data were centred and scaled before performing these algorithms. For the distance-based approaches, clusters were determined based on Euclidean distances.

**Latent Class Analysis**

Latent class analysis (LCA) assumes that the data represent a mixture of distribution that can be separated based on unobserved (latent) categorical variables or classes.^4^ Viewed simplistically, LCA is a probabilistic, model-based, approach to identifying subgroups. A maximum likelihood framework is used to optimize parameters of the model in order to reveal and characterize the observed latent classes. Next, probabilities are generated for each observation to belong in each of the classes in the model. In the case of continuous variables, the parameters are means and standard deviations of the underlying distributions, whereas, categorical variables are parameterized with thresholds. For these analyses, continuous variables were log-transformed where appropriate and full information maximum likelihood (FIML) was used to deal with missing data. FIML uses all the available data, complete and incomplete, to estimate model parameters, such that the patterns of incompleteness may be informative of model characteristics. Five models, comprising 1-5 classes respectively, were fit to each dataset. The models were run repeatedly with multiple starting seeds as the initial parameter setting. To avoid local maxima as algorithmic solutions, models were only considered for analysis if the maximum likelihood was repeated 20 times among the permutation of starting seeds. Up to a maximum of 1000 random starts were used to seek the minimum number of repetitions of the maximum likelihood. The optimal number of classes were determined by comparing Bayesian Information Criteria (BIC), entropy, and the Vuong-Lo-Mendell-Rubin test across models. Once the model comprising the optimal number of classes was determined, each observation was assigned to the class for which it had the highest probability to belonging.

**Consensus Clustering**

Distance based clustering methods are highly sensitive to small algorithmic changes such as the random starting position (k-means) or when two different pairs of clusters are nearly tied for the merge (hierarchical). To obtain more consistent results and minimize the risk of random discoveries, we used an analytic approach called consensus clustering.^5^ In consensus clustering, each clustering algorithm is run repeatedly (in this analysis 1000 times), where each run subsamples variables and observation from the data. Where applicable, unique random starting locations are used for each run. Clustering solutions from individual runs are accumulated into a consensus matrix. Within this matrix, pairwise scores are generated for the frequency with which each given pair is assigned to the same cluster among the numerous runs in the consensus matrix (a measure of inter-run consistency). The optimal number of clusters can then be determined by calculating the proportion of ambiguous clustering (PAC) using the cumulative distribution function (CDF) plot of the consensus index value. In the CDF plot, the bottom left signifies points that are never or rarely together (value approaching 0), while the top right signifies points always being clustered together (value approaching 1). A CDF plot with steep slopes at the 0 and 1 ends with a flat plateau in between represents a desirable clustering solution. In this analysis, differences in PAC between the clustering solutions for an algorithm in each dataset was used to determine the optimal number of clusters that best fit the data. Further, heatmaps of the consensus matrix were also constructed to visualize cluster separation. To avoid investigator subjectivity in the process of selecting the optimal number of clusters, an automated function to determine the lowest PAC value was used. This method was applied in the analyses using k-means, Partitioning Around Medoids, Hierarchical Clustering, and Spectral Clustering. We used the Bioconductor *R package “ConsensusClusterPlus.”* Additionally, for spectral clustering we used the *R package “kernlab.”*

**K-Means**

The k-means algorithm aims to partition the data into multiple number (k) of groups where each observation is assigned to the closest group centre. The algorithm proceeds by first randomly initializing k centres (centroids). Distances between each centroid and each point (observation) are calculated. Based on this distance metric, points are assigned to the closest centroid. Next, for each centroid, a new centre is calculated using the mean of all the point assigned to the centroid. The algorithm then repeats the above procedure of point reassignment based on new centroids and the process is repeated until no further changes in cluster assignments are observed.

**Partitioning Around Medoids (PAM)**

In principle, PAM is similar to k-means, however, an actual data point (observation) from the dataset, rather than the mean of all points within a cluster, serves as the centre (known as medoid). For each cluster, the initial starting point in PAM is also randomly determined and the observations that are the closest distance to these medoids are assigned to it. Next, the current medoid is swapped with each non-medoid data points and the quality of the swap, as evaluated by a dissimilarity index, is compared with the original medoid. If the dissimilarities are reduced with the swap, then the new data-point becomes the medoid and closest points are assigned to it. The procedure is repeated until the dissimilarity is no longer minimized and the medoid at the point is used to determine the cluster for each observation. Theoretically, PAM is less sensitive to outliers compared to k-means and as the medoid for each cluster is an actual data point, it facilitates easier characterization of clusters based on the cluster centre.

**Hierarchical Clustering (HC)**

HC uses distances to build trees of clusters of observations that are similar within, and dissimilar, across clusters. HC can be performed as either bottom up or top down. For these analyses, a bottom up approach, also referred to as agglomerative HC, was used. Agglomerative HC begins with each observation forming its own cluster, and at each subsequent step, the two closest / most similar clusters are merged until all observations are included within one cluster forming a tree (graphically known as a dendrogram) that represent the different joins and where each join level contains a unique number of clusters. We used Euclidean distance as the basic metric of distance between observations. We used Ward’s method as the function to evaluate cluster proximity for merging at each step. Ward’s method, also known as minimum variance method, is defined as the difference between total within cluster sum of squares for clustering separately (Ci and Cj) and within cluster sum of squares for combination (Cij), such that at each step the clusters with the smallest inter-cluster distance are merged and minimizes intra-cluster variance.

**Spectral**

Spectral clustering is a density-based algorithm rooted in graph-based evaluation, such that each data point is treated as a graph-node and clusters are grouped together according to similarity. Spectral clustering first reduces dimensionality of multidimensional data and seeks clusters in the reduced dimensional space. The algorithm begins by building an undirected graph in which each point is represented by a node. Two nodes are said to have a connecting edge if they are k-nearest neighbours of one another. The graph is then converted to an adjacency matrix (A) in which a 1 is placed at location (i,j) and (j,i) if there is an edge between node i and j and is a matrix that represent similarity of pairwise nodes. A degree matrix (D) is also calculated in which the i^th^ diagonal entry represents the number of edges connecting the i^th^ node and represents the interconnectivity of the points. Graph Laplacian matrix (L) is constructed and defined as L = D-A. From this matrix, for the purposes of dimension reduction, Eigen values and Eigen vectors are calculated and stacked into a matrix. to determine k and the cluster segmentation respectively. The main advantage of spectral clustering is that it makes no assumptions about the structural dimensionality of the clusters, whereas, more traditional approaches such as K-means assume an organised (predominantly spherical) structure around the centroid. Spectral, therefore, allows identification of clusters that need not be uniformly distributed and the final clustering solution is more likely to be representative of the inherent data structure.

**Supervised**

Supervised clustering methods optimize directly on the differential treatment response yielding individualized treatment effect predictions for each observation. Specifically, models were trained to predict directionality of treatment responses with treatment allocation and outcome groups serving as the dependent variables. For each observation, a coefficient of treatment effect on outcome was generated (positive indicating harm and negative indicating benefit). Out-of-sample estimations were performed to reduce bias and overfitting. Two clusters were determined by partitioning the observations into directionality of the coefficients.

**Model Based Recursive Partitioning (MOB)**

MOB is a recursive partitioning decision tree model that splits the data into clusters according to those that have homogeneous treatment responses. The tree is partitioned using independent (predictor) variables to identify splits that lead to nodes where outcome responses to treatment are most divergent. Rather than the clusters being based simply on the outcome variables, MOB uses the intercepts and / or slope of treatment effect as a determining parameter for node splitting. At each split locally, specific variables are used to partition the tree, and parameter instability is tested to evaluate whether split parameter is locally well fit. The algorithm splits the node using the covariates with the highest split instability and repeated at each node until further splits no longer lead to parameter instability. Once the tree is completed, an associated logistic regression model is fit to the terminal node and is applied to predict the treatment response for each observation.^6^ The analyses for MOB were performed using the *R package “partykit.”*

**Causal Forest (CF)**

Causal forest are causal inference algorithms that use independent (predictor) variables to seek heterogeneous responses to treatment.^7^ Viewed simply, causal forests are similar to random forest in that they are both ensemble tree-based algorithms. In random forest, each tree splits the data to maximize predictive accuracy, whereas, causal forest splits data to maximize heterogeneity across the split. In these analyses, the probability of death given treatment is the dependent variable, conditioned on the partitioning variables in order to most accurately: 1) represent true treatment effect for a given observation and 2) the variability of treatment response across a population. For each observation, multiple trees are added to the model such that each tree uses a randomly selected subset of covariates to construct the trees. For each observation individualized treatment effect is estimated from aggregating all the trees predicting a treatment effect for that observation. All models comprised 10000 trees and autotuning initialized. The *R package “grf”* was used for the analysis.

**X-learner Random Forest (XL_RF) and Bayesian additive regression trees (BART)**

The x-learner algorithms aim to estimate the conditional average treatment effect (CATE) for observations.^8^ These algorithms estimate CATE under both treatment and control conditions using observed outcomes. The difference between the two estimates of CATE for an individual observation is used to predict the individual treatment effect. Specifically, the algorithm first creates two models using the predictor variables: one predicting death in control group ${(\mu}_{0})$ and the other in the treatment group ${(\mu}_{1})$. These predictor models are then applied to the opposite group (e.g. control model is applied to obtain predictions for treatment group). Next, treatment effects are modelled separately (residuals $\hat{\tau}_{1} and \hat{\tau}_{0}$ from these predictions are compared to the true outcome). Third, a propensity score (g(x)) accounts for the probability of being treated. Finally, treatment effect is estimated based on the following weighted average:

$$\tau\left( x \right)=g\left( x \right)* \hat{\tau}_{0}+(1-g\left( x \right)*\hat{\tau}_{1})$$

The R package “soerenkuenzel/causalToolbox” on github was used for these analyses. Within the x-learner framework, two separate algorithms were implemented using 1) random forest (RF) and 2) Bayesian additive regression trees (BART) as the modeling framework. RF models are an ensemble of decision trees classifying patients based on Boolean (“yes / no”) groups for the outcome. A random selection of variables is used for each split of each classification tree. The aggregate predictions of all the trees are used to determine the final classification prediction for an observation. BART models use a boosting approach where each prediction is the sum of weak learner built iteratively to improve prediction from prior trees in the model. However, instead of multiplying each sequential tree by the learning rate, a regularization prior is put on the model parameters, thereby limiting the risks of overfitting. For computational ease, in both RF and BART, default parameter settings of the R package were used.

**Variable Importance and Seed Instability**

Clustering algorithms do not easily detail which variables are most important in the clustering. In order to study the variables that were most influential for each set of clustering assignment, XGBoost models were built to predict the clusters. For the XGBoost models, the cluster assignments were the dependent variables and all partitioning variables were independent variables. XGBoost is an adaptation of a gradient boosted machine algorithm in which multiple weak learners are combined into a strong learner. Specifically, sequential trees are built to correct errors made in prior trees similar to the algorithmic approach of BART. The gain in classification accuracy associated with each variable across the numerous tress in the XGBoost model is aggregated to calculate a variable’s ranking as the most important. These analyses were performed using the *R package “xgboost.”*

To assess the stability of each algorithm the clusters were re-derived using 10 different random initiating seeds. The treatment by cluster interaction p-value and odds ratios were compared across runs (**Figure S2a-c**) along with the adjusted rand index (ARI) (**Figure S2d**). ARI measures cluster overlap between two sets of cluster assignments. The metric ranges from -1 to 1 where 1 is complete agreement, 0 is expected agreement based on chance, and -1 is no agreement (even less than expected by change). ARI can be thought of as the metric for cluster agreement in this context and a value closer to 1 is desirable.

**References:**

1. Brower RG, Lanken PN, MacIntyre N, et al. Higher versus lower positive end-expiratory pressures in patients with the acute respiratory distress syndrome. *N Engl J Med* 2004; **351**(4): 327-36.

2. National Heart L, Blood Institute Acute Respiratory Distress Syndrome Clinical Trials N, Wiedemann HP, et al. Comparison of two fluid-management strategies in acute lung injury. *N Engl J Med* 2006; **354**(24): 2564-75.

3. National Heart L, Blood Institute ACTN, Truwit JD, et al. Rosuvastatin for sepsis-associated acute respiratory distress syndrome. *N Engl J Med* 2014; **370**(23): 2191-200.

4. Sinha P, Calfee CS, Delucchi KL. Practitioner's Guide to Latent Class Analysis: Methodological Considerations and Common Pitfalls. *Crit Care Med* 2021; **49**(1): e63-e79.

5. Wilkerson MD, Hayes DN. ConsensusClusterPlus: a class discovery tool with confidence assessments and item tracking. *Bioinformatics* 2010; **26**(12): 1572-3.

6. Seibold H, Zeileis A, Hothorn T. Model-Based Recursive Partitioning for Subgroup Analyses. *Int J Biostat* 2016; **12**(1): 45-63.

7. Athey S, Wager S. Estimating Treatment Effects with Causal Forests: An Application. *arXiv: Methodology* 2019.

8. Kunzel SR, Sekhon JS, Bickel PJ, Yu B. Metalearners for estimating heterogeneous treatment effects using machine learning. *Proc Natl Acad Sci U S A* 2019; **116**(10): 4156-65.

**Figure Legend:**

**Figure S1: Alluvial plot depicting the overlap of patients from clusters in one algorithm to clusters in a second algorithm among pairs of clustering algorithms where heterogeneity of treatment effect (HTE) was detectable.** ALVEOLI = Assessment of Low Tidal Volume and Elevated End-Expiratory Pressure to Obviate Lung Injury, FACTT = Fluids and Catheters Treatment Trial, SAILS = Statins for Acutely Injured Lungs from Sepsis. LCA = Latent class analysis, PAM = partitioning around medoids, HC = Hierarchical clustering, MOB = model based recursive partitioning, CF = Causal forest, XL-RF = X-learner with Random Forest (RF); XL-BART = Bayesian Additive Regression Trees (BART). OR = Odds ratio for heterogeneity of treatment effect > 1 was associated with harm with the randomised intervention (H = Harm, B = benefit).

**Figure S2: Random seed sensitivity to identifying clusters with significant heterogeneity of treatment effect and cluster reproducibility. For each algorithm in each trial, 10 separate runs were tested with a new random seed on each run** (where missing data required imputation, the seeds were also changed for the imputation algorithm)**.** LCA = Latent class analysis, PAM = partitioning around medoids, HC = Hierarchical clustering, MOB = model based recursive partitioning, CF = Causal forest, XL-RF = X-learner with Random Forest (RF); XL-BART = Bayesian Additive Regression Trees (BART). **Panel S2a: Odds ratio for heterogeneity of treatment effect in clusters for each algorithm in the Assessment of Low Tidal Volume and Elevated End-Expiratory Pressure to Obviate Lung Injury (ALVEOLI) trial** (OR > 1 was associate with harm)**. Panel S2b: Odds ratio for heterogeneity of treatment effect in clusters for each algorithm in the Fluids and Catheters Treatment (FACTT) Trial** (OR > 1 was associate with harm)**. Panel S2c: Odds ratio for heterogeneity of treatment effect in clusters for each algorithm in the Statins for Acutely Injured Lungs from Sepsis (SAILS) Trial** (OR > 1 was associate with harm)**.**

**Tables**

**Table S1.** Baseline characteristics of predictor variables used for clustering in the three randomised controlled trials (** these variables, including outcome variables, were not used for clustering).

|  | | | ALVEOLI (n = 549) | FACCT (n = 1000) | SAILS (n = 745) |
| --- | --- | --- | --- | --- | --- |
| Study Period (years)** | |  | 1999-2003 | 2000-2005 | 2010-2013 |
| Sex | | Female (n) | 302 (55%) | 534 (53%) | 380 (51%) |
| Male (n) | | | 247 (45%) | 466 (47%) | 365 (49%) |
| Race | | White | 412 (75%) | 641 (64%) | 590 (79%) |
| Other | | | 137 (25%) | 359 (36%) | 155 (21%) |
| Age (years) | | | 51 ± 17 | 50 ± 16 | 54 ± 16 |
| Body mass index (kg/m^2^) | | | 27.4 ± 6.9 | 28.6 ± 7.5 | 30.7 ± 10 |
| Temperature (°C) | | | 38.4 ± 1.0 | 38.4 ± 1.0 | 38.1 ± 1.0 |
| SBP (mmHg) | | | 88 ± 17 | 88 ± 17 | 85 ± 16 |
| Heart rate (bpm) | | | 125 ± 24 | 125 ± 22 | 118 ± 23 |
| Urine Output (L/24 hours) | | | 1.8 (1.2 – 3.0) | 1.7 (1.1 – 2.7) |  |
| PaO_2_/FiO_2_ ratio | | | 128 ± 58 | 132 ± 63 | 139 ± 64 |
| Tidal Volume (mL) | | | 511 ± 118 | 465 ± 109 | 413 ± 87 |
| Minute Ventilation (L/min) | | | 12.4 ± 3.8 | 11.9 ± 4.0 | 10.8 ± 3.2 |
| PEEP (cm H_2_O) | | | 10 (5 - 12) | 10 (5 - 12) | 10 (5 - 11) |
| PaCO_2_ (mmHg) | | | 38.7 ± 9.1 | 40.4 ± 10.1 | 40.3 ± 10.8 |
| Respiratory rate (breath/min^-1^) | | | 32 (25-38) | 35 (28-40) | 32 (27 - 38) |
| Haematocrit (%) | | | 30 ± 6 | 30 ± 7 | 30 ± 6 |
| WBC (10^3^/µL) | | | 14.5 ± 10 | 14.8 ± 13 | 15.7 ± 12.3 |
| Platelets (10^3^/µL) | | | 177 ± 124 | 195 ± 126 | 186 ± 125 |
| Sodium (mmol/L) | | | 137 ± 5 | 138 ± 6 | 138 ± 5 |
| Creatinine (mg/dL) | | | 1.8 ± 2.0 | 1.4 ± 0.9 | 1.53 ± 1.1 |
| Glucose (mg/dL) | | | 133 ± 64 | 125 ± 59 | 125 ± 49 |
| Albumin (g/dL) | | | 2.1 ± 0.6 | 2.2 ± 0.7 | 2.2 ± 0.6 |
| Bilirubin (mg/dL) | | | 1.6 ± 3.2 | 1.6 ± 2.7 | 1.3 ± 1.8 |
| Bicarbonate (mmol/L) | | | 21.7 ± 5.5 | 21.2 ± 5.6 | 21.8 ± 5.5 |
| ARDS Risk Factors: | | Trauma | 45 (8%) | 74 (7%) | 6 (1%) |
| Sepsis | | | 120 (22%) | 233 (23%) | 145 (19%) |
|  | Aspiration | | 84 (15%) | 149 (15%) | 49 (7%) |
| Pneumonia | | | 221 (40%) | 471 (47%) | 529 (71%) |
| Other | | | 79 (15%) | 73 (7%) | 16 (2%) |
| Vasopressor at Enrolment (n) | | | 144 (26%) | 327 (33%) | 406 (55%) |
| Interleukin-6 (pg/mL) | | | 244 (94 – 742) | 132 (47 – 413) | 443 (173 – 1510) |
| Interleukin-8 (pg/mL) | | | 40 (16 – 98) | 32 (16 – 78) | 53 (26 – 135) |
| Soluble TNF receptor-1 (pg/mL) | | | 4277 (2605 – 8448) | 4021 (2591 – 6806) | 5347 (3084 – 8857) |
| ICAM-1 (ng/mL) | | | 926 (605 – 1385) | 1385 (851 – 2228) | 358 (236 – 508) |
| Protein C (% control) | | | 87 ± 53 | 96 ± 60 | 81 ± 42 |
| PAI-1 (ng/mL) | | | 60 (30 – 144) | 67 (46 – 101) | 4 (2 – 9) |
| Von Willebrand Factor (% control)^a^ | | | 395 (247 – 624) | 165 (96 – 278) | -- |
| Surfactant Protein-D (ng/mL)^a^ | | | 101 (50 – 217) | 135 (63 – 281) | -- |
| Ventilator Free Days** | | | 18 (0 - 24) | 17 (0 - 23) | 20 (0 - 25) |
| Mortality at 90 days** | | | 148 (27%) | 284 (28%) | 204 (27%) |

ALVEOLI = Assessment of Low Tidal Volume and Elevated End-Expiratory Pressure to Obviate Lung Injury, FACTT = Fluids and Catheters Treatment Trial, SAILS = Statins for Acutely Injured Lungs from Sepsis, TNF = Tumour necrosis factor, ICAM = Intercellular adhesion molecule, PAI = Plasminogen activator inhibitor. a = Variables were not measure in the SAILS (Statins for Acutely Injured Lungs from Sepsis) trial. SBP = Systolic blood pressure, PEEP = Positive end-expiratory pressure, WBC = White blood cell count, ICAM-1 = Intercellular adhesion molecule-1, PAI-1 = Plasminogen activator inhibitor-1.

**Table S2**. Major inclusion and exclusion criteria for the three randomised controlled trials used for secondary analyses to evaluate the clustering algorithms.

| **Major inclusion criteria common in all three RCTs** |
| --- |
| Intubation and mechanical ventilation |
| PaO_2_/FiO_2_ Ratio < 300 mmHg |
| Recent bilateral infiltrates on chest radiograph |
| Absence of evidence of left atrial hypertension |
| **Major inclusion criteria specific to FACTT** |
| Central venous catheter in situ or intention of clinician to place a one |
| **Major inclusion criteria specific to SAILS** |
| Known or suspected infection and either of the following criteria for a systemic inflammatory response: white-cell count > 12,000/mm^3^ or < 4000/mm^3^, or a differential count > 10% band, or core body temperature > 38°C or < 36°C |
| **Timing of ARDS in ALVEOLI** |
| < 36 hours elapsed since meeting eligibility criteria |
| **Timing of ARDS in FACTT and SAILS** |
| Presence of ARDS < 48 hours |
| **Major exclusion criteria common to all three RCTs** |
| 6-month mortality rate greater than 50 percent |
| **Major exclusion criteria specific to FACTT** |
| Presence of a pulmonary-artery catheter after the onset of acute lung injury |
| **Major exclusion criteria specific to SAILS** |
| Serum creatine kinase, aspartate aminotransferase, or alanine aminotransferase > 5-times upper limit of normal |
| Statin use within 48 hours of meeting eligibility criteria |

*RCT =* Randomised Controlled Trial, *ALVEOLI = Assessment of Low Tidal Volume and Elevated End-Expiratory Pressure to Obviate Lung Injury, FACTT = Fluids and Catheters Treatment Trial, SAILS = Statins for Acutely Injured Lungs from Sepsis.*

**Table S3. Missing observations in the three randomised controlled trial for all predictor variables used in the clustering algorithms**

|  | ALVEOLI | FACCT | SAILS |
| --- | --- | --- | --- |
| Number of patients | 549 | 1000 | 745 |
| Gender | 0 | 0 | 0 |
| Race | 0 | 0 | 0 |
| Body mass index | 44 | 84 | 2 |
| ARDS Risk Factors | 0 | 0 | 0 |
| Temperature | 1 | 2 | 0 |
| Heart rate | 1 | 3 | 0 |
| Systolic blood pressure | 1 | 2 | 0 |
| Respiratory rate | 1 | 2 | 4 |
| Urine output | 20 | 26 | 4 |
| Vasopressor at Enrollment | 0 | 0 | 1 |
| PaO_2_/FiO_2_ ratio | 1 | 0 | 0 |
| PaCO_2_ | 25 | 84 | 12 |
| Minute Ventilation | 5 | 27 | 43 |
| Tidal Volume | 42 | 121 | 213 |
| Positive End-Expiratory Pressure | 3 | 4 | 22 |
| Mean Airway Pressure | 29 | 117 | 316 |
| Haematocrit | 1 | 25 | 1 |
| WBC | 4 | 13 | 1 |
| Platelets | 5 | 8 | 0 |
| Sodium | 1 | 3 | 0 |
| Glucose | 4 | 20 | 1 |
| Creatinine | 2 | 5 | 1 |
| Bicarbonate | 1 | 26 | 5 |
| Albumin | 35 | 220 | 96 |
| Bilirubin | 33 | 268 | 92 |
| Interleukin-6 | 28 | 116 | 25 |
| Interleukin-8 | 21 | 116 | 25 |
| Soluble tumour-necrosis factor receptor-1 | 20 | 83 | 25 |
| Intercellular adhesion molecule-1 | 20 | 159 | 25 |
| Protein C | 21 | 159 | 25 |
| Plasminogen activator inhibitor-1 | 24 | 83 | 25 |
| Von Willebrand Factor | 20 | 69 | NA |
| Surfactant Protein-D | 39 | 116 | NA |

ALVEOLI = Assessment of Low Tidal Volume and Elevated End-Expiratory Pressure to Obviate Lung Injury, FACTT = Fluids and Catheters Treatment Trial, SAILS = Statins for Acutely Injured Lungs from Sepsis.
