# Supplementary material for: Comparison of machine learning clustering algorithms for detecting heterogeneity of treatment effect in acute respiratory distress syndrome: A secondary analysis of three randomised controlled trials
Source: eBioMedicine. 2021 Dec 1;74:103697. doi: 10.1016/j.ebiom.2021.103697 (PMC8645454; doi:10.1016/j.ebiom.2021.103697)
Supplement: Supplementary file 2 [file mmc2.pdf]

a

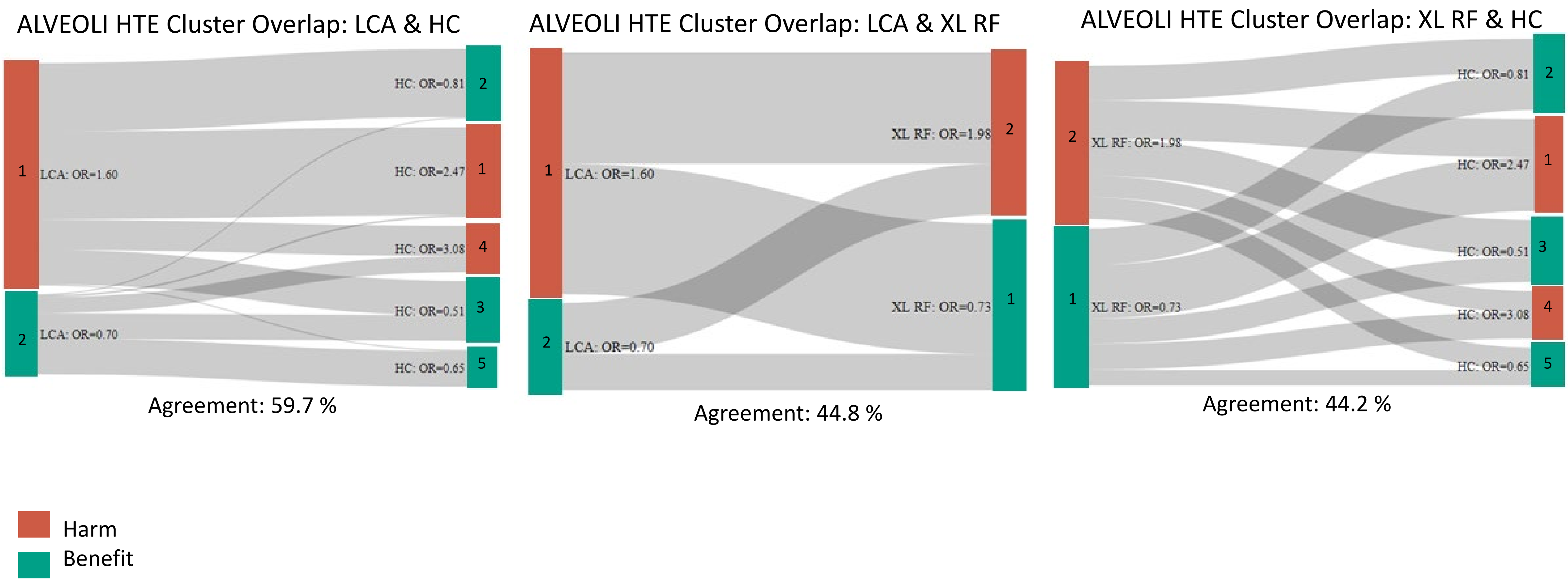
ALVEOLI HTE Cluster Overlap: LCA & HC
1 LCA: OR=1.60
2 LCA: OR=0.70
HC: OR=0.81 2
HC: OR=2.47 1
HC: OR=3.08 4
HC: OR=0.51 3
HC: OR=0.65 5
Agreement: 59.7 %
ALVEOLI HTE Cluster Overlap: LCA & XL RF
1 LCA: OR=1.60
2 LCA: OR=0.70
XL RF: OR=1.98 2
XL RF: OR=0.73 1
Agreement: 44.8 %
ALVEOLI HTE Cluster Overlap: XL RF & HC
2 XL RF: OR=1.98
1 XL RF: OR=0.73
HC: OR=0.81 2
HC: OR=2.47 1
HC: OR=0.51 3
HC: OR=3.08 4
HC: OR=0.65 5
Agreement: 44.2 %
Harm
Benefit

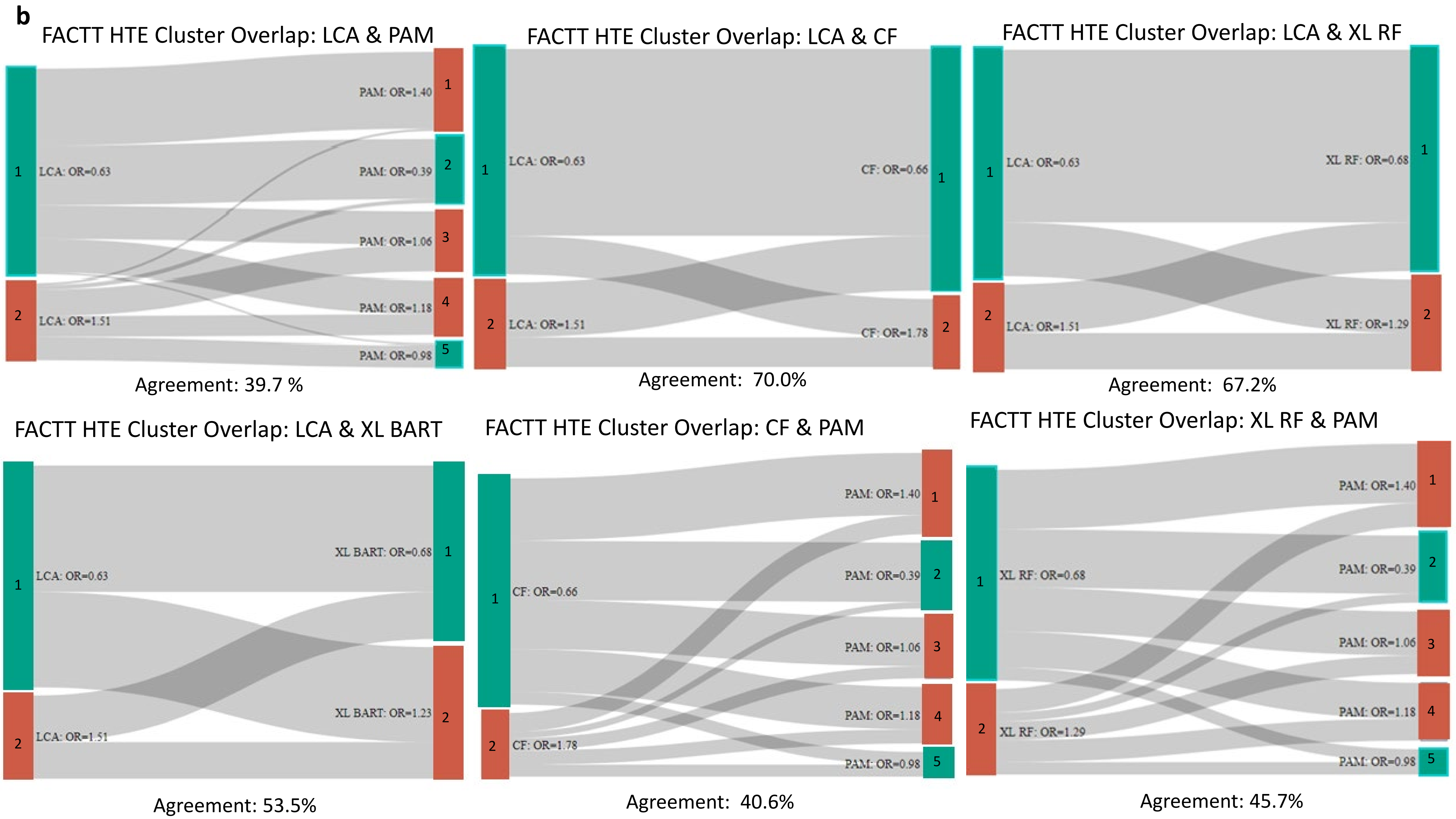
b
FACTT HTE Cluster Overlap: LCA & PAM
1
LCA: OR=0.63
2
LCA: OR=1.51
PAM: OR=1.40
1
PAM: OR=0.39
2
PAM: OR=1.06
3
PAM: OR=1.18
4
PAM: OR=0.98
5
Agreement: 39.7 %
FACTT HTE Cluster Overlap: LCA & CF
1
LCA: OR=0.63
2
LCA: OR=1.51
CF: OR=0.66
1
CF: OR=1.78
2
Agreement: 70.0%
FACTT HTE Cluster Overlap: LCA & XL RF
1
LCA: OR=0.63
2
LCA: OR=1.51
XL RF: OR=0.68
1
XL RF: OR=1.29
2
Agreement: 67.2%
FACTT HTE Cluster Overlap: LCA & XL BART
1
LCA: OR=0.63
2
LCA: OR=1.51
XL BART: OR=0.68
1
XL BART: OR=1.23
2
Agreement: 53.5%
FACTT HTE Cluster Overlap: CF & PAM
1
CF: OR=0.66
2
CF: OR=1.78
PAM: OR=1.40
1
PAM: OR=0.39
2
PAM: OR=1.06
3
PAM: OR=1.18
4
PAM: OR=0.98
5
Agreement: 40.6%
FACTT HTE Cluster Overlap: XL RF & PAM
1
XL RF: OR=0.68
2
XL RF: OR=1.29
PAM: OR=1.40
1
PAM: OR=0.39
2
PAM: OR=1.06
3
PAM: OR=1.18
4
PAM: OR=0.98
5
Agreement: 45.7%

c

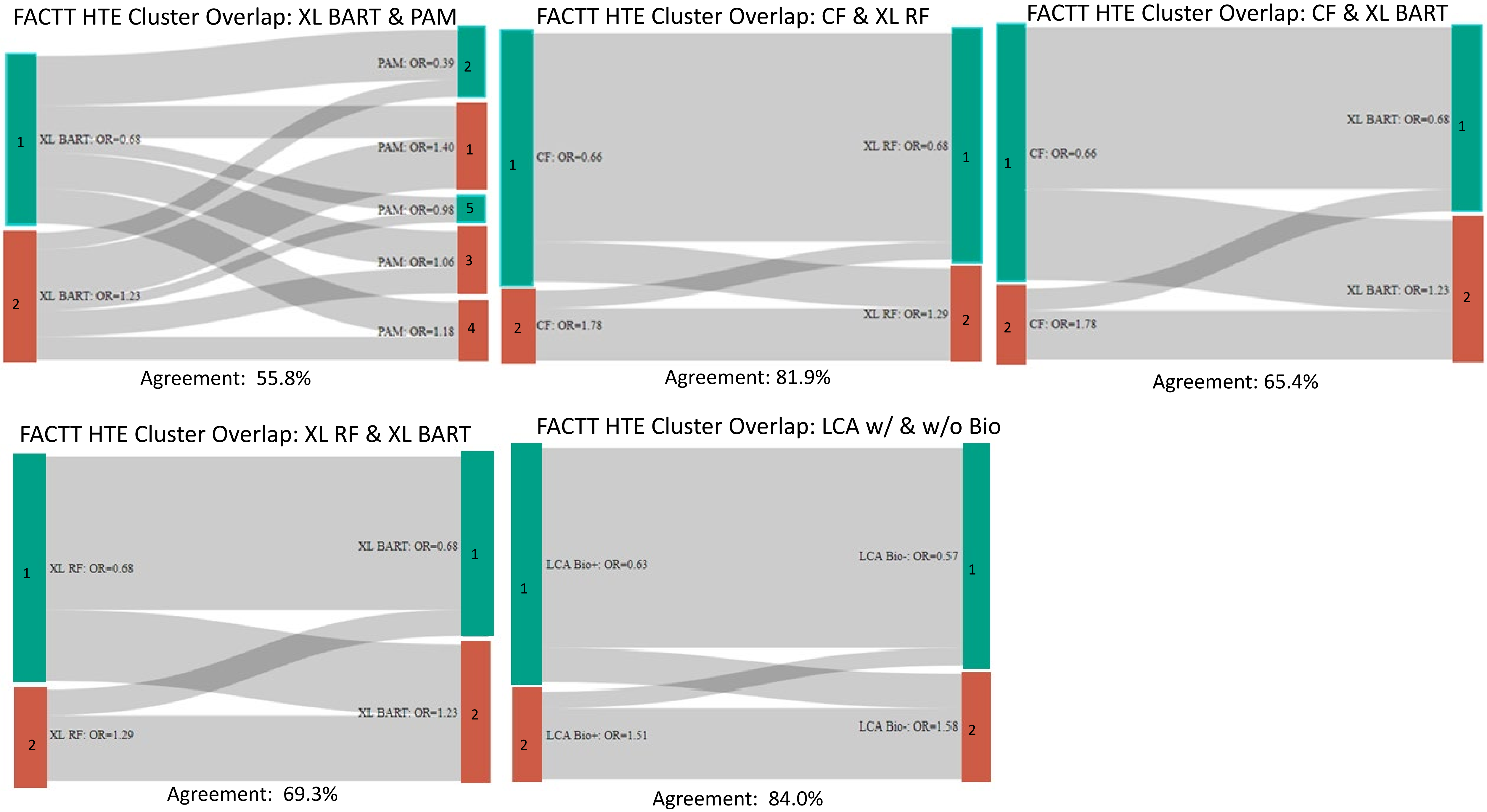
FACTT HTE Cluster Overlap: XL BART & PAM
1
XL BART: OR=0.68
2
XL BART: OR=1.23
PAM: OR=0.39
2
PAM: OR=1.40
1
PAM: OR=0.98
5
PAM: OR=1.06
3
PAM: OR=1.18
4
Agreement: 55.8%
FACTT HTE Cluster Overlap: CF & XL RF
1
CF: OR=0.66
2
CF: OR=1.78
XL RF: OR=0.68
1
XL RF: OR=1.29
2
Agreement: 81.9%
FACTT HTE Cluster Overlap: CF & XL BART
1
CF: OR=0.66
2
CF: OR=1.78
XL BART: OR=0.68
1
XL BART: OR=1.23
2
Agreement: 65.4%
FACTT HTE Cluster Overlap: XL RF & XL BART
1
XL RF: OR=0.68
2
XL RF: OR=1.29
XL BART: OR=0.68
1
XL BART: OR=1.23
2
Agreement: 69.3%
FACTT HTE Cluster Overlap: LCA w/ & w/o Bio
1
LCA Bio+: OR=0.63
2
LCA Bio+: OR=1.51
LCA Bio-: OR=0.57
1
LCA Bio-: OR=1.58
2
Agreement: 84.0%

d

SAILS HTE Cluster Overlap: Kmeans & CF

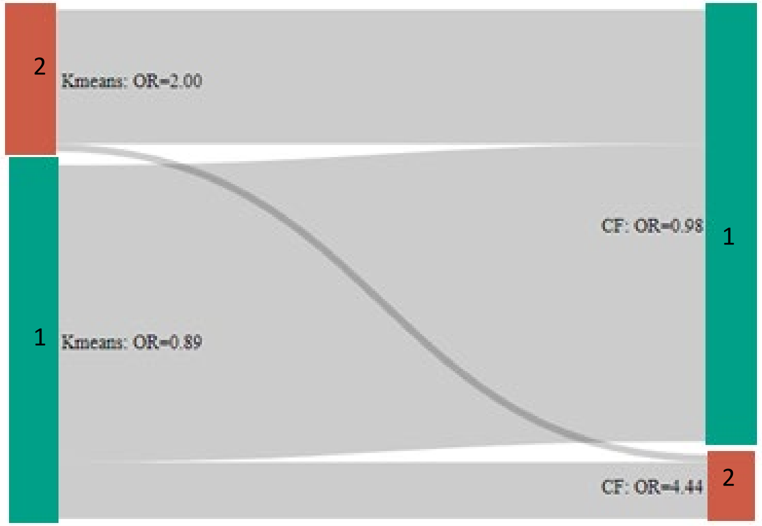

Agreement: 61.5 %
