# Supplementary material for: Comparison of machine learning clustering algorithms for detecting heterogeneity of treatment effect in acute respiratory distress syndrome: A secondary analysis of three randomised controlled trials
Source: eBioMedicine. 2021 Dec 1;74:103697. doi: 10.1016/j.ebiom.2021.103697 (PMC8645454; doi:10.1016/j.ebiom.2021.103697)
Supplement: Supplementary file 3 [file mmc3.pdf]

a

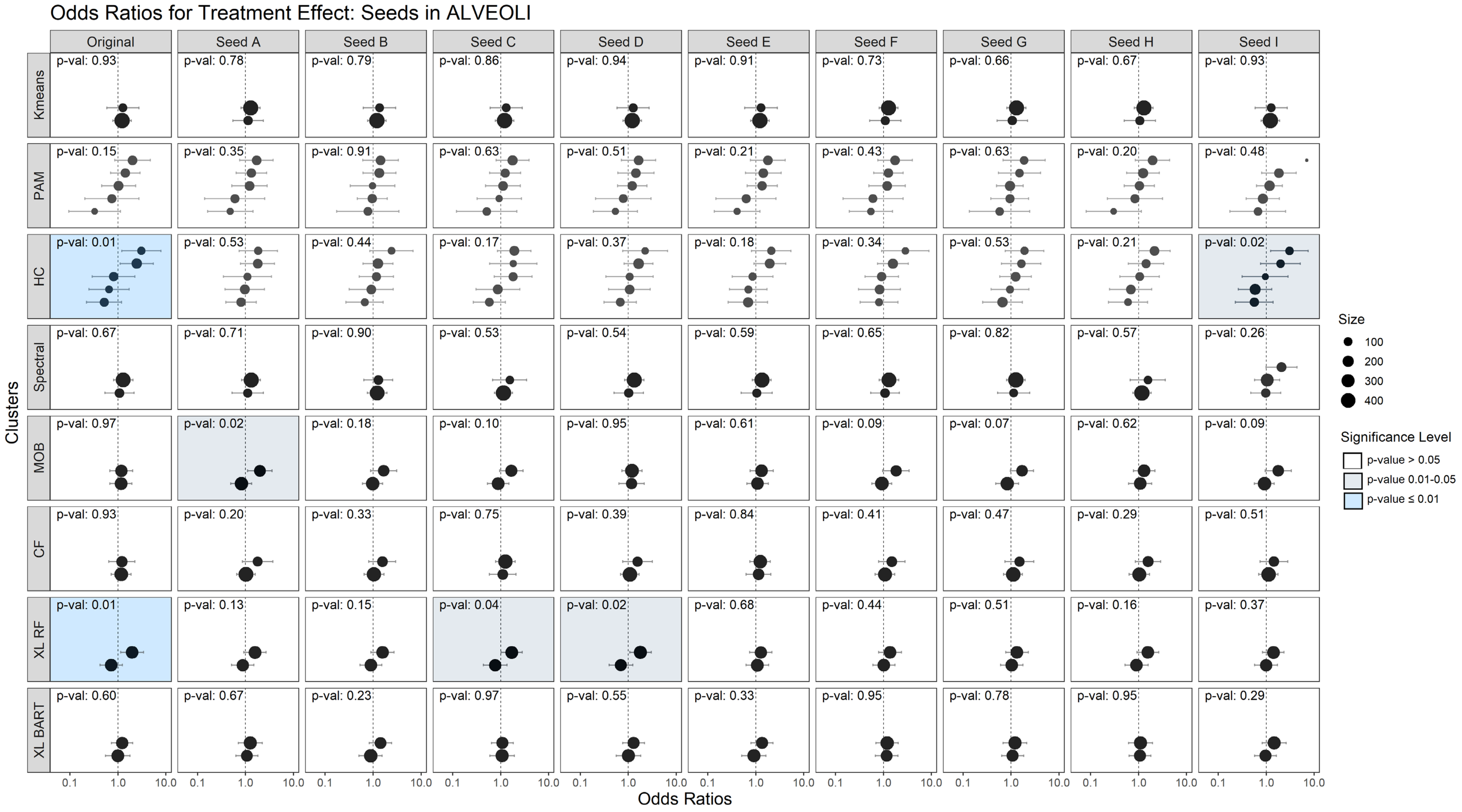
Odds Ratios for Treatment Effect: Seeds in ALVEOLI
Original
Seed A
Seed B
Seed C
Seed D
Seed E
Seed F
Seed G
Seed H
Seed I
Kmeans
p-val: 0.93 p-val: 0.78 p-val: 0.79 p-val: 0.86 p-val: 0.94 p-val: 0.91 p-val: 0.73 p-val: 0.66 p-val: 0.67 p-val: 0.93
PAM
p-val: 0.15 p-val: 0.35 p-val: 0.91 p-val: 0.63 p-val: 0.51 p-val: 0.21 p-val: 0.43 p-val: 0.63 p-val: 0.20 p-val: 0.48
HC
p-val: 0.01 p-val: 0.53 p-val: 0.44 p-val: 0.17 p-val: 0.37 p-val: 0.18 p-val: 0.34 p-val: 0.53 p-val: 0.21 p-val: 0.02
Spectral
p-val: 0.67 p-val: 0.71 p-val: 0.90 p-val: 0.53 p-val: 0.54 p-val: 0.59 p-val: 0.65 p-val: 0.82 p-val: 0.57 p-val: 0.26
MOB
p-val: 0.97 p-val: 0.02 p-val: 0.18 p-val: 0.10 p-val: 0.95 p-val: 0.61 p-val: 0.09 p-val: 0.07 p-val: 0.62 p-val: 0.09
CF
p-val: 0.93 p-val: 0.20 p-val: 0.33 p-val: 0.75 p-val: 0.39 p-val: 0.84 p-val: 0.41 p-val: 0.47 p-val: 0.29 p-val: 0.51
XL RF
p-val: 0.01 p-val: 0.13 p-val: 0.15 p-val: 0.04 p-val: 0.02 p-val: 0.68 p-val: 0.44 p-val: 0.51 p-val: 0.16 p-val: 0.37
XL BART
p-val: 0.60 p-val: 0.67 p-val: 0.23 p-val: 0.97 p-val: 0.55 p-val: 0.33 p-val: 0.95 p-val: 0.78 p-val: 0.95 p-val: 0.29
Clusters
0.1 1.0 10.0
Odds Ratios
Size
100
200
300
400
Significance Level
p-value > 0.05
p-value 0.01-0.05
p-value ≤ 0.01

b

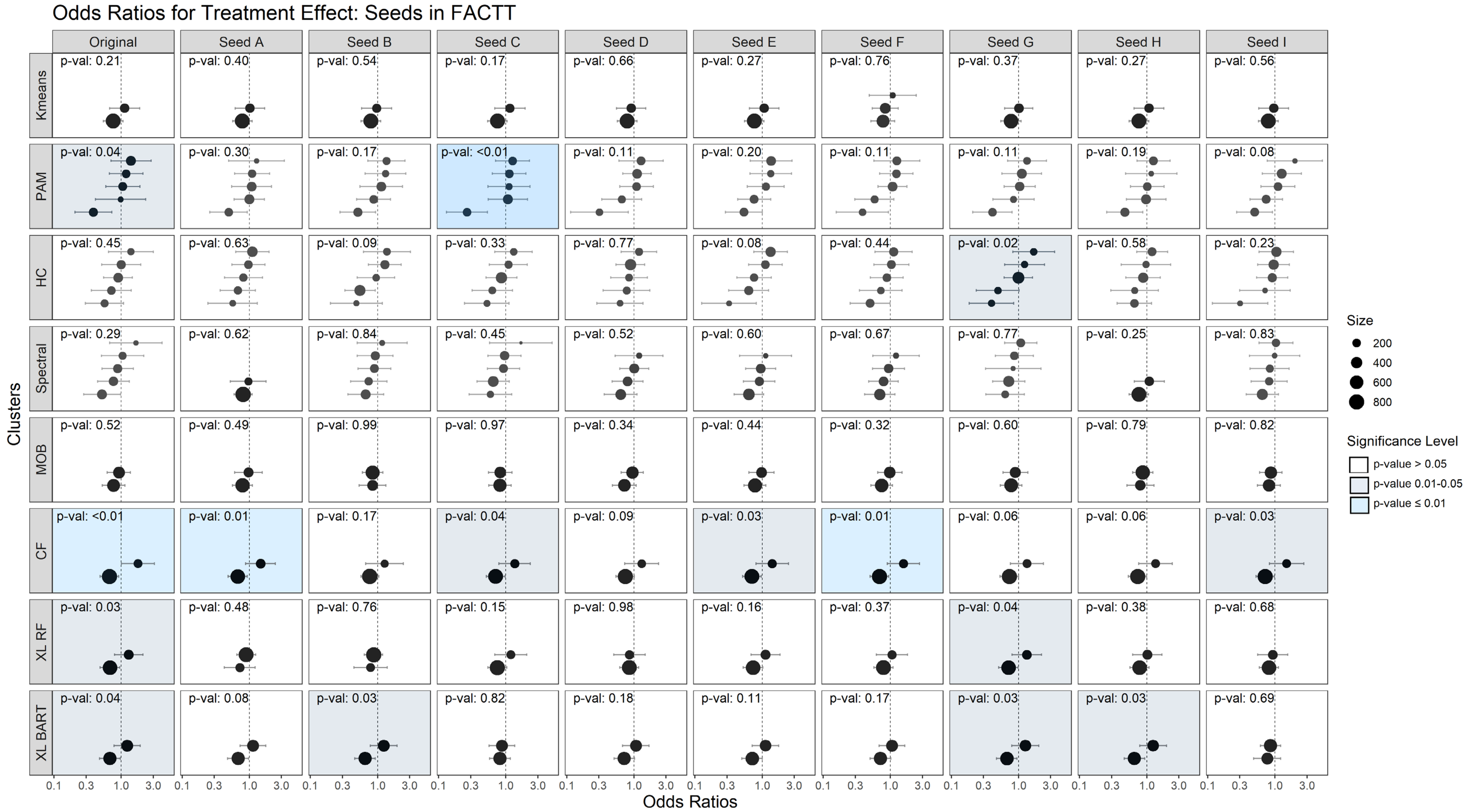

Odds Ratios for Treatment Effect: Seeds in FACTT
Original
Seed A
Seed B
Seed C
Seed D
Seed E
Seed F
Seed G
Seed H
Seed I
Kmeans
p-val: 0.21
p-val: 0.40
p-val: 0.54
p-val: 0.17
p-val: 0.66
p-val: 0.27
p-val: 0.76
p-val: 0.37
p-val: 0.27
p-val: 0.56
PAM
p-val: 0.04
p-val: 0.30
p-val: 0.17
p-val: <0.01
p-val: 0.11
p-val: 0.20
p-val: 0.11
p-val: 0.11
p-val: 0.19
p-val: 0.08
HC
p-val: 0.45
p-val: 0.63
p-val: 0.09
p-val: 0.33
p-val: 0.77
p-val: 0.08
p-val: 0.44
p-val: 0.02
p-val: 0.58
p-val: 0.23
Spectral
p-val: 0.29
p-val: 0.62
p-val: 0.84
p-val: 0.45
p-val: 0.52
p-val: 0.60
p-val: 0.67
p-val: 0.77
p-val: 0.25
p-val: 0.83
MOB
p-val: 0.52
p-val: 0.49
p-val: 0.99
p-val: 0.97
p-val: 0.34
p-val: 0.44
p-val: 0.32
p-val: 0.60
p-val: 0.79
p-val: 0.82
CF
p-val: <0.01
p-val: 0.01
p-val: 0.17
p-val: 0.04
p-val: 0.09
p-val: 0.03
p-val: 0.01
p-val: 0.06
p-val: 0.06
p-val: 0.03
XL RF
p-val: 0.03
p-val: 0.48
p-val: 0.76
p-val: 0.15
p-val: 0.98
p-val: 0.16
p-val: 0.37
p-val: 0.04
p-val: 0.38
p-val: 0.68
XL BART
p-val: 0.04
p-val: 0.08
p-val: 0.03
p-val: 0.82
p-val: 0.18
p-val: 0.11
p-val: 0.17
p-val: 0.03
p-val: 0.03
p-val: 0.69
Clusters
0.1 0.3 1.0 3.0
Odds Ratios
Size
200
400
600
800
Significance Level
p-value > 0.05
p-value 0.01-0.05
p-value ≤ 0.01

c

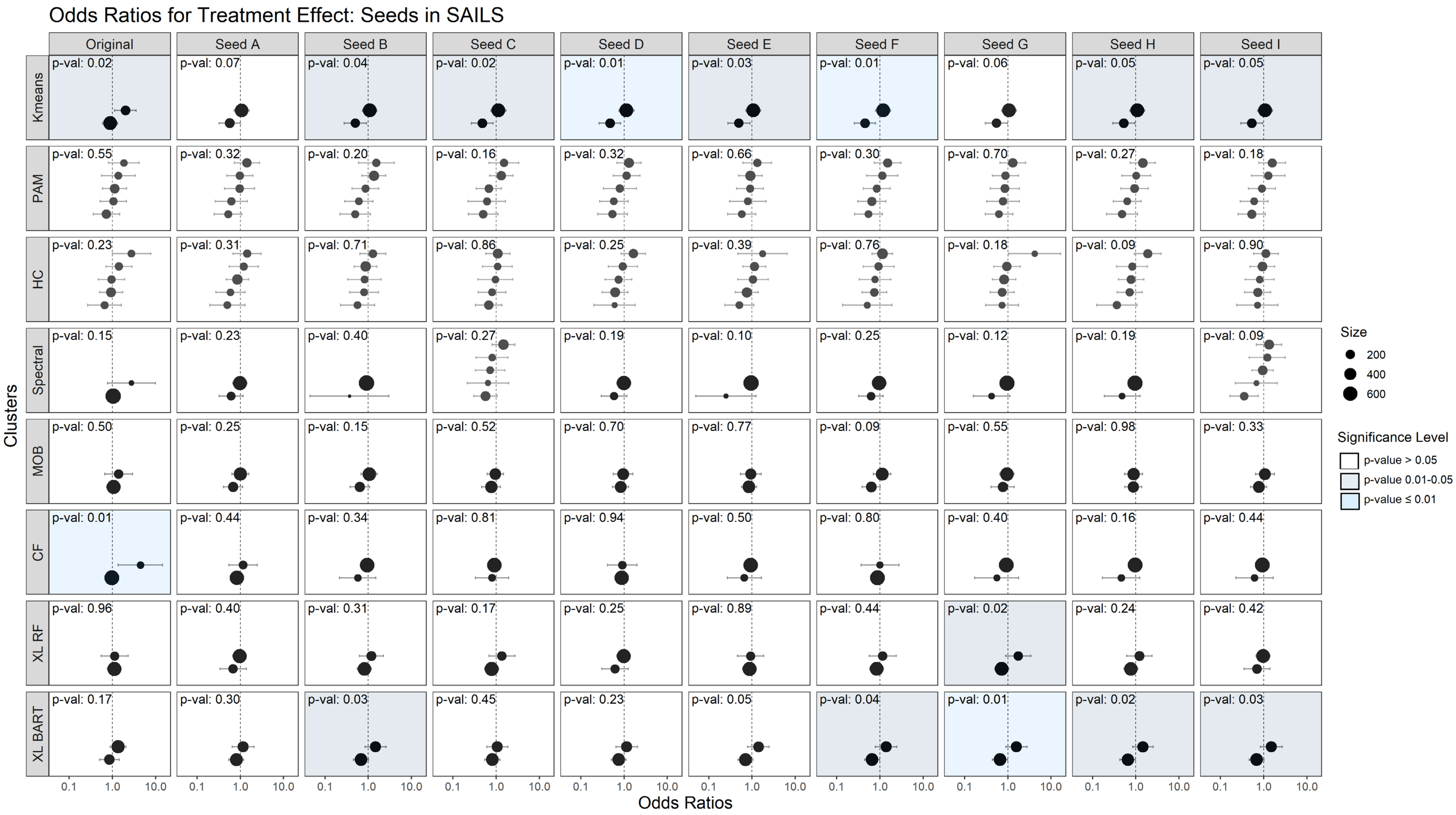

Odds Ratios for Treatment Effect: Seeds in SAILS
Original
Seed A
Seed B
Seed C
Seed D
Seed E
Seed F
Seed G
Seed H
Seed I
Kmeans
p-val: 0.02
p-val: 0.07
p-val: 0.04
p-val: 0.02
p-val: 0.01
p-val: 0.03
p-val: 0.01
p-val: 0.06
p-val: 0.05
p-val: 0.05
PAM
p-val: 0.55
p-val: 0.32
p-val: 0.20
p-val: 0.16
p-val: 0.32
p-val: 0.66
p-val: 0.30
p-val: 0.70
p-val: 0.27
p-val: 0.18
HC
p-val: 0.23
p-val: 0.31
p-val: 0.71
p-val: 0.86
p-val: 0.25
p-val: 0.39
p-val: 0.76
p-val: 0.18
p-val: 0.09
p-val: 0.90
Spectral
p-val: 0.15
p-val: 0.23
p-val: 0.40
p-val: 0.27
p-val: 0.19
p-val: 0.10
p-val: 0.25
p-val: 0.12
p-val: 0.19
p-val: 0.09
MOB
p-val: 0.50
p-val: 0.25
p-val: 0.15
p-val: 0.52
p-val: 0.70
p-val: 0.77
p-val: 0.09
p-val: 0.55
p-val: 0.98
p-val: 0.33
CF
p-val: 0.01
p-val: 0.44
p-val: 0.34
p-val: 0.81
p-val: 0.94
p-val: 0.50
p-val: 0.80
p-val: 0.40
p-val: 0.16
p-val: 0.44
XL RF
p-val: 0.96
p-val: 0.40
p-val: 0.31
p-val: 0.17
p-val: 0.25
p-val: 0.89
p-val: 0.44
p-val: 0.02
p-val: 0.24
p-val: 0.42
XL BART
p-val: 0.17
p-val: 0.30
p-val: 0.03
p-val: 0.45
p-val: 0.23
p-val: 0.05
p-val: 0.04
p-val: 0.01
p-val: 0.02
p-val: 0.03
Clusters
0.1
1.0
10.0
Odds Ratios
Size
200
400
600
Significance Level
p-value > 0.05
p-value 0.01-0.05
p-value ≤ 0.01
